# Inflammation–Nature's Way to Efficiently Respond to All Types of Challenges: Implications for Understanding and Managing “the Epidemic” of Chronic Diseases

**DOI:** 10.3389/fmed.2018.00316

**Published:** 2018-11-27

**Authors:** Jeanette M. Bennett, Glenn Reeves, George E. Billman, Joachim P. Sturmberg

**Affiliations:** ^1^Department of Psychological Science, StressWAVES Biobehavioral Research Lab, The University of North Carolina at Charlotte, Charlotte, NC, United States; ^2^School of Biomedical Sciences and Pharmacy, Faculty of Health and Medicine, University of Newcastle, Newcastle, NSW, Australia; ^3^Department of Physiology and Cell Biology, Dorothy M. Davis Heart and Lung Research Institute, The Ohio State University, Columbus, OH, United States; ^4^School of Medicine and Public Health, Faculty of Health and Medicine, University of Newcastle, Newcastle, NSW, Australia; ^5^Foundation President, International Society for Systems and Complexity Sciences for Health, Delaware, United States

**Keywords:** chronic disease, inflammation, interdisciplinary, clinical care, stress, anti-inflammatory lifestyle, complex adaptive systems

## Abstract

Siloed or singular system approach to disease management is common practice, developing out of traditional medical school education. Textbooks of medicine describe a huge number of discrete diseases, usually in a systematic fashion following headings like etiology, pathology, investigations, differential diagnoses, and management. This approach suggests that the body has a multitude of ways to respond to harmful incidences. However, physiology and systems biology provide evidence that there is a *simple* mechanism behind this phenotypical variability. Regardless if an injury or change was caused by trauma, infection, non-communicable disease, autoimmune disorders, or stress, the typical physiological response is: an increase in blood supply to the area, an increase in white cells into the affected tissue, an increase in phagocytic activity to remove the offending agent, followed by a down-regulation of these mechanisms resulting in healing. The *cascade of inflammation* is the body‘s unique mechanism to maintain its integrity in response to macroscopic as well as microscopic injuries. We hypothesize that chronic disease development and progression are linked to uncontrolled or dysfunctional inflammation to injuries regardless of their nature, physical, environmental, or psychological. Thus, we *aim to reframe* the prevailing approach of management of individual diseases into a more integrated systemic approach of treating the “person as a whole,” enhancing the patient experience, ability to a make necessary changes, and maximize overall health and well-being. The first part of the paper reviews the local immune cascades of pro- and anti-inflammatory regulation and the interconnected feedback loops with neural and psychological pathways. The second part emphasizes one of nature's principles at work—*system design* and *efficiency*. Continually overwhelming this finely tuned system will result in systemic inflammation allowing chronic diseases to *emerge*; the pathways of several common conditions are described in detail. The final part of the paper considers the implications of these understandings for clinical care and explore how this lens could shape the physician-patient encounter and health system redesign. We conclude that healthcare professionals must advocate for an anti-inflammatory lifestyle at the patient level as well as at the local and national levels to enhance population health and well-being.

## Overview of the immune system

The primary objective of the immune system is to rid the body of foreign or non-self-cellular material, such as bacteria, viruses, fungi, parasites, and damaged cells. There are two branches that make up the immune system: the innate and adaptive branches (Table 1). Although each branch has distinctly different responsibilities in preserving the integrity of the body, they work in concert to most efficiently remove harmful antigens (non-self-cellular matter) from the body. The innate immune system is a rudimentary first line of defense and is responsible for initiating the inflammatory response. The adaptive immune system is more highly evolved and designed to “learn” and create “memory” as the organism is exposed to antigen throughout its life. Both branches are responsible for monitoring the entire body for antigen presences; however, activation of each branch requires different mechanisms.

**Table 1:** Characteristic functions of the immune system by branch.

	**Innate immune system**	**Adaptive immune system**
Self/non-self-discrimination	Absent, reaction is against evolutionarily-conserved foreign patterns	Present, reaction is against foreign structures presented to specific immune cells
Lag phase	Absent, response is immediate	Present, response takes at least a few days
Specificity	Limited, the same response is mounted to a wide variety of agents	High, the response is directed only to the agents that initiated it
Diversity	Limited, hence limited specificity	Extensive, and resulting in a wide range of immune responses
Memory	Absent, subsequent exposures to the same agent generate the same response	Present, subsequent exposures to the same agent induce amplified responses
Internal effectors	Phagocytes, NK cells, antimicrobial proteins, lipoproteins, complement, inflammation, fever	Humoral (B cells) and cellular (T cells) immunity

Primary lymph organs include the bone marrow and thymus, where lymphocytes are generated and/or differentiated. For example, T cells differentiate in the thymus and undergo positive and negative selection, allowing T cells into the bloodstream that will be activated only when recognizing a cell with major histocompatibility complex (MHC) markers and presenting an antigen. Secondary lymph organs consist of the lymph nodes and spleen, which are reservoirs of naive immune cells. Lymph nodes filter the lymphatic system and provide the interaction between antigen-activated leukocytes and B- and T-cells. Naive leukocytes monitor blood and tissue for foreign antigens and/or are recruited to a site of inflammation by immune messengers such as cytokines to become activated. Once activated, leukocytes clear the affected area of foreign antigen, infected, injured or dead cells, and aid in repair.

Immune cells use cytokines to communicate as autocrine, paracrine, or endocrine messengers between one another and with other biological systems resulting in synergistic, antagonistic, or multiple effects. The cytokine environment can modulate the adaptive immune response. For example, the production of interleukin 2 (IL-2) and interferon-gamma (IFN-γ) support the activation of cytotoxic T-lymphocytes and are necessary to fight off bacteria and viruses. However, high levels of IL-4 and IL-6 support activation and proliferation of B-lymphocytes favoring antibody development.

### Innate immune system

The innate immune system not only involves cellular defenses, but also physical and chemical barriers. Physical barriers include the skin and mucous membranes of the respiratory and gastrointestinal system. For example, a chemical barrier would be the acidic pH of the stomach. Although the innate immune system is simpler, it is responsible for the immediate, non-specific inflammation such as the warmth, redness, pain, and swelling associated with a cut on the skin. Using pattern recognition receptors (PRRs) that identify common membrane ligands on bacteria, the innate immune system can detect a wide range of microbial antigens and instigate an inflammatory response. In addition, soluble pattern recognition receptors are found in the blood, including the complement system. Complement is a cascade of proteins that results in holes being “punched” into the membrane of microorganisms and aids in the recruitment of inflammatory cells.

The immune cells actively involved in the innate immune response include macrophages (activated monocytes), neutrophils, natural killer (NK) cells, and dendritic cells (DCs). Macrophages, neutrophils and DCs use phagocytosis to clear antigen or microbes and produce reactive oxygen species to kill microbes. NK cells lyse virally infected cells. Once activated, NK cells continue to recruit new immune cells via cytokines. Activated DCs and macrophages, also known as antigen presenting cells (APCs), migrate to lymph nodes to elicit activation of the adaptive immune system. Cytokines critical to the effectiveness of the innate immune system include IL-1, IL-6, tumor necrosis factor-alpha (TNF-α), and IFN-α. These immune messengers induce fever, pain, and fatigue, clinically known as *sickness behavior* (1, 2), and activate the hypothalamic-pituitary-adrenal axis (HPA-axis) (3). Recently, additional endogenous biological markers such as insulin, cholesterol, and lipoproteins have been identified as playing an important role in innate immune system function (4).

### Adaptive immune system

The more evolved adaptive immune system can only be found in vertebrates and is responsible for increasing the intensity and specificity of antigen clearance as well as developing memory to allow for a faster removal of antigen during a secondary exposure. In humans, the adaptive response takes 10–14 days to mount a primary antigen specific response. See Table 1 for a comparison of the characteristic functions for each immune system branch.

Two major types of T cells, T helper (T_H_) cells (CD4+), and T cytotoxic (T_C_) cells (CD8+), circulate throughout the blood and lymphatic system and reside in the secondary lymph organs (5). Once the T cells are presented with antigen via an APC, they differentiate into memory and effector cells. T_C_ effector cells are cytotoxic T lymphocytes (CTL) and lyse the antigen-bearing cells. T_H_ effector cells are responsible for cytokine production and directing B cell development or mobilization. The current cytokine environment produced by innate immune cells influences the development of T_H_ cells, which in turn impacts the course of the immune response to favor one of two major directions (6). T_H_1 cells produce cytokines IL-2, IFN-γ, and TNF-α, which drive CTL activation and supports cellular immunity. This cell-mediated immune response is critical to mounting an effective response against intracellular bacteria and viruses. On the other hand, T_H_2 cells produce IL-4 and IL-5; in turn supporting B cell activation and differentiation also known as humoral immunity (7). T_H_ and T_C_ memory cells monitor the body via the blood and lymphatic systems for recurrent exposure to antigen. Both cell types are critical in mounting a fast, efficient secondary response to an antigen. Additional T cell subsets have also been defined as playing a critical role in host defense, and further T cell subsets are increasingly recognized as being important: T regulators (T_REG_) and T_H_17 cells. T_REG_ cells play a central role in the regulation of other immune responses, releasing TGF-β and IL-10 to down-modulate excessive activation of T_H_1 and T_H_2 cells that, if left unchecked, can predispose to autoimmune or allergic conditions, respectively. T_H_17, as their name suggests, release IL-17, and are important in antimicrobial defenses involving neutrophil recruitment, especially to mucous membranes, playing a particularly important role in defense against certain specific pathogens such as candida.

The humoral immune response is important in battling extracellular microbes and mounting an antibody response during primary and secondary responses to antigen. Immature B cells travel throughout the body and are found in high concentration in the lymph nodes. Activated B cells become effector plasma cells and manufacture antigen-specific antibodies that neutralize free antigen or mark infected cells for destruction. Memory B cells are long lasting cells and support the faster clearance during the 2nd exposure via creating larger targets for phagocytic or lytic immune cells to attack as in the primary response. For example, the antibody complexes are detected by NK cells and macrophages, leading to the phagocytosis of the antibody-neutralized antigen or infected cell. In addition, antibodies can activate or perpetuate the complement cascade.

### Inflammation—A universal response

#### General principles

Inflammation begins when the body senses “danger,” in the form of infective, traumatic, ischemic, physical, chemical, or other challenges. Exposure to pathogen-associated molecular patterns (PAMPs) and damage-associated molecular patterns (DAMPs) leads to activation of cells from the monocyte-macrophage lineage, resulting in expression of pro-inflammatory and suppression of anti-inflammatory genes (8). Production of cytokines, chemokines and other chemicals mediates the non-specific cellular recruitment and humorally-mediated vascular changes (9). These cellular processes are mediated by various intracellular signaling and nuclear transcription pathways, especially: NF-κB and AP-1 (driving pro-inflammatory cytokines such as TNF-α and IL-1, plus expression of chemokines [e.g., CCL2, CXCL8] and endothelial adhesion molecules); and IRF (interferon-response factors) 3 and 7 (which promote production of Type I interferons (IFN-α and β, important for antiviral defense) (10).

Additionally, non-antigenic stimuli may be perceived as “danger” and initiate an inflammatory response; these influences include trauma such as radiation, ischaemia, toxin exposure, and even psychological stress. These factors may induce inflammation by various mechanisms, including activation of neuroendocrine pathways (as seen with emotional stress, see section Depression), or through the upregulation of “stress” pathways which stimulate inflammatory signaling, as seen with microglial activation in ischemic stroke (11). Environmental toxins and chemicals (e.g., pesticide residues, additives, preservatives) may also induce inflammatory activity through activation of cytochrome P450 pathways, inducing reactive oxygen species (12), or metabolism to reactive intermediates and neoantigens with immunotoxic effects (13). A noteworthy example of this convergence of environmental stimuli and inflammatory responses is being revealed by study of the aryl hydrocarbon receptor [AhR, (14)]. AhR activation can mediate inflammatory responses to a range of endogenous and exogenous ligands, and plays a key role in mucosal immunity and normal barrier function, as well as acting as a pathogenic pathway for various oncogenic factors, such as dioxin (15).

**Figure 1:**
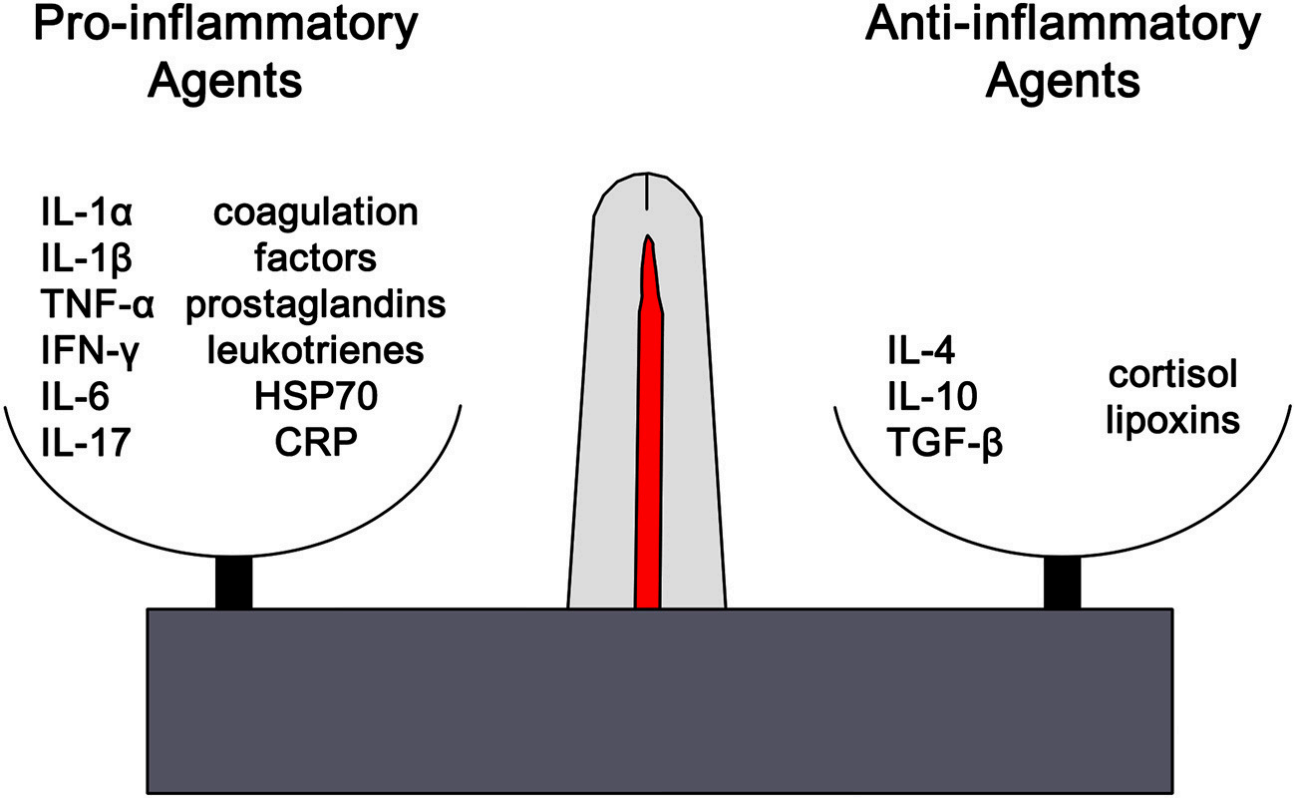
The outcome of any inflammatory response is dictated by the balance between pro-inflammatory and anti-inflammatory factors. Each of these opposing pathways is mediated by different cytokine and hormonal influences. For example, inflammation is favored by IL-1β, IL-6, and TNF-α, whilst being inhibited by IL-10 and TGF-β. The distinctions are not absolute and can vary based on the context. However, excess or chronic inflammation is seen in conditions where the mechanisms mediating homeostasis and balance between the two pathways become compromised. IL1α, interleukin-1alpha; IL-1β, interleukin-1beta; TNF-α, tumor necrosis factor-1alpha; IFN-γ, interferon-gamma; IL-6, interleukin-6; IL-17, interleukin-17; HSP70, heat shock protein 70, CRP, C-reactive protein; IL-4, interleukin-4, IL-1, interleukin-1, TGF-β, transforming growth factor-beta.

Clinically inflammation is recognized by the classical findings of dolor (pain), calor (heat), rubor (redness), tumor (swelling), and *functio laesa* (loss of function). Tissue injury results in the release of histamine which stimulates capillary dilation, resulting in vascular stasis allowing the migration of phagocytes and plasma leakage (redness, heat, and swelling). Release of bradykinin increases pain sensitivity in tissues containing nerve endings. Loss of function is regarded as a neurological reflex in response to pain. Phagocytic activity removes pathogens and the down-regulation of the inflammatory cascade results in healing. Inflammation is favored by factors such as IL-1, IL-6, and TNF-α, whilst being inhibited by cytokines including IL-10 and TGF-β. The distinctions are not absolute, however, and some factors play dual roles. For example, IL-4 interferes with inflammation in some tumors, but favors antibody production in allergies (Figure 1). In the context of chronic inflammation, IL-6 is often deemed pro-inflammatory, however, there are examples of when IL-6 can have anti-inflammatory affects (16) as well as may indicate other natural ongoing processes such as tissue repair (17). Thus, context is critical when examining inflammation levels to ensure proper conclusions are being drawn.

These “macroscopic” changes of inflammation are governed by immune-regulatory pathways. However, the release of immune mediators and cytokines as a consequence of the immune response trigger neuronal responses that amplify the local responses to inflammation and also trigger systemic neuroendocrine and neural responses that finally result in resolution of the process and restoration of the normal homeostatic state (18). These normal feedback loops can be interrupted by prolonged or inappropriate central nervous system activation, resulting in either excessive inflammation by inadequate hormonal suppression or uncontrolled infection by excessive or prolonged anti-inflammatory responses (19).

**Figure 2:**
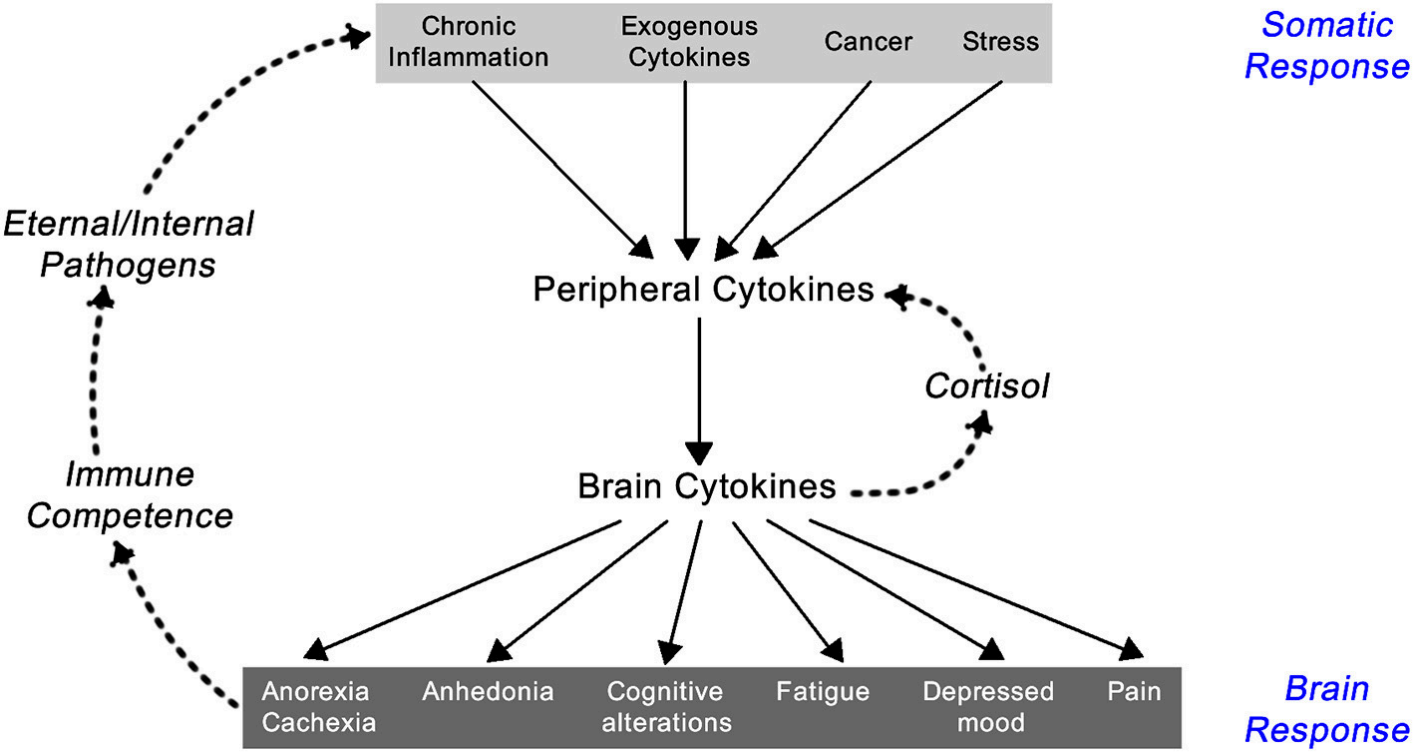
Feedback loop between the somatic and central nervous system components of the inflammatory response [exogenous cytokines are typically synthetically made cytokines used as treatment for a variety of immune-related diseases like IFN-α for hepatitis C infection or IL-2 for renal cell carcinoma; adapted from Dantzer (1)].

Inflammation results in the systemic responses of feeling ill—being feverish, nauseated, and off one's food, tired but also suffering from fragmented sleep, irritable, and low in mood, having poor concentration and being forgetful, and showing social withdrawal. These symptoms are triggered by pro-inflammatory cytokines including IL-1α, IL-1β, TNF-α, IFN-γ, and IL-6. Though they are a local response to an infection they stimulate the brain-cytokine system resulting in the experience of illness symptoms also known as *sickness behavior*, and prompt reduced activity and rest so as to better cope with the infection (1) (Figure 2). In addition, non-cytokine mediators of the inflammatory balance include pro-inflammatory chemicals such as CXCL8 chemokines and certain metalloproteinases, along with anti-inflammatory agents including antimicrobial peptides, TIMP (tissue inhibitor of metalloproteinases), and chemokine CCL2 (20, 21).

The brain has immune cells such as microglia, macrophages, and dendritic cells that in response to inflammatory stimuli can produce cytokines and prostaglandins that can stimulate neural and non-neural brain cell receptors. The brain also monitors peripheral immune responses by afferent nerve stimulation, humoral pathways, cytokine exchange across the blood-brain barrier, and IL-1 receptor activation on perivascular macrophages and endothelial cells of brain venules (22).

Anti-inflammatory cytokines IL-10 and TGF-rβ in the brain ameliorate the sickness experience. In health, there is a balance between pro- and anti-inflammatory cytokines in the brain. Since aging is associated with increased activity of the innate immune system the brain produces a larger amount of pro-inflammatory cytokines but a decreased production of anti-inflammatory cytokines resulting in more pronounced *sickness behavior* (22).

#### Inflammation as a *whole body* response

Inflammation *triggers a whole body response* by activation of many different feedback loops (19). The central nervous system (CNS) reacts rapidly to environmental stimuli, resulting in the binding of neurotransmitters, and neuropeptides to the same signaling pathways stimulated by immune mediators. Immune modulators released at the site of inflammation interact with neurotransmitter receptors of the pain pathways, and in turn, local neuropeptides can release pro-inflammatory mediators like histamine to enhance the local inflammatory response.

The neural response to inflammation is rapid, but varies over time, and can have an amplifying or dampening effect on the inflammatory process, and thus the clinically observed *behavior*s of disease over time. Overall, these neural response patterns aim to maintain normal physiological homeostasis in response to immune system stimulation and the restoration of normal tissue function.

**Figure 3:**
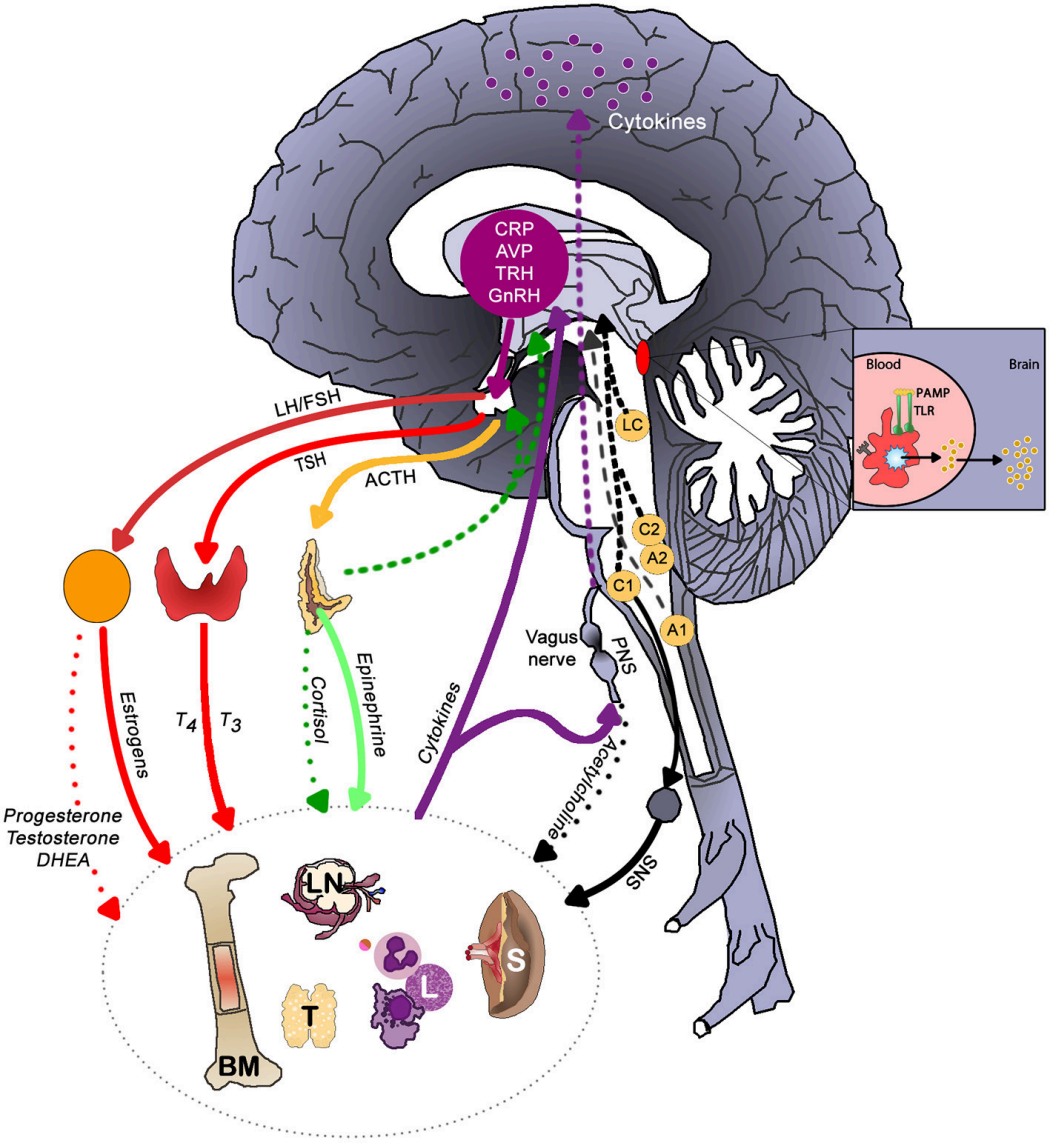
The main brain-immune system pathways and feedback loops illustrating the interconnected effects of physical and emotional stress in health. Sympathetic nervous system (SNS) activation facilitates immune cell activity and systemic immune responses, while the parasympathetic nervous system (PNS) and the hypothalamic-pituitary-adrenal (HPA) axis generally inhibit inflammatory responses. In a well-regulated system, cortisol provides negative feedback to the HPA axis. Chronic activation of the stress response systems can lead to excessive immune cell activity and promote system inflammation due to the reduced activity of cholinergic anti-inflammatory pathway and development of glucocorticoid insensitivity. Cytokines regulate autonomic nervous system function and the HPA axis as well as induce sickness behaviors. The brain receives information about systemic inflammation levels via the afferent vagal nerve and “leaky” portions of the blood brain barrier especially near the circumventricular organs and these messages influence cytokine production within the brain. Often elevated systemic inflammation increases glia production of cytokines. CRP, C-reactive protein; AVP, arginine vasopressin; TRH, thyrotropin-releasing hormone; GnRH, gonadotrophin-releasing hormone; LH, luteinizing hormone; FSH, follicle stimulating hormone; TSH, thyroid stimulating hormone; PAMP, pathogen-associated molecular pattern; TLR, toll-like receptor; LC, locus coeruleus; C1 & C2, adrenergic cell groups in brainstem; A1& A2, noradrenergic cell groups in brainstem; T4, tetraiodothyronine; T3, triioidothyronine; DHEA, dehydroepiandrosterone; SNS, sympathetic nervous system; PNS, parasympathetic nervous system; BM, bone marrow; LN, lymph node; T, thymus; L, lymphocytes; S, spleen. Dashed lines represent feedback on the brain. In the periphery, solid lines indicate activation, whereas dotted lines represent inhibition.

Figure 3 illustrates the main brain-immune system pathways and feedback loops. Sympathetic nervous system (SNS) activation facilitates immune cell activity and systemic immune responses, while the parasympathetic nervous system (PNS) and the hypothalamic-pituitary-adrenal (HPA) axis generally inhibit inflammatory responses. However, chronic activation of the stress response systems can lead to excessive immune cell activity and promote systemic inflammation (details discussed in next section).

Immune cells contain the required receptors to respond to neurotransmitters, neuropeptides and neurohormones and their signaling pathways. Microglia and neurons can respond to peripheral cytokine production. Furthermore, microglia, the immune system's resident neural cells, are sensitive to bacterial lipopolysaccharides (LPS), triggering CNS inflammation directly without the involvement of peripheral cytokines due to expression of toll-like receptors (TLRs). The aged brain, whether associated with chronological age or chronic disease state, has more reactive microglia that have an exaggerated cytokine response to stimulation and disrupts neural plasticity as well as *behavioral* and *cognitive* function (23). Thus, inflammation whether peripheral or neural in nature dramatically influences the functioning of the whole body.

##### The pivotal role of the hypothalamic-pituitary-adrenal (HPA)-axis

Cortisol, a glucocorticoid, influences daily bodily functions such as energy utilization/storage, memory formation, respiration, heart rate, gastrointestinal function, and mood. It has a stable diurnal rhythm, but can also be released in response to internal (e.g., excessive immune activity) and external (e.g., perceived threat) stressors. Cortisol is the end product of the hypothalamic-pituitary-adrenal (HPA) axis. Corticotropin releasing hormone (CRH) from the hypothalamus initiates the release of adrenocorticotropic hormone (ACTH) from the anterior pituitary. ACTH travels via the blood stream and stimulates the adrenal cortex to produce cortisol (24). Via negative feedback on glucocorticoid receptors in the hippocampus, cortisol stops the further release of CRH and ACTH (25). Various other hormones including androgens, estrogens, and posterior pituitary hormones, vasopressin and oxytocin can modulate the *HPA-axis* (26–29).

In a well-regulated system, immune cells express glucocorticoid receptors; allowing cortisol to inhibit immune cell activation and pro-inflammatory cytokine release (30, 31). However, chronic stress may elevate cortisol levels for an extended time, leading to the downregulation of glucocorticoid receptor expression (32–34). As a result, unregulated immune cells can generate excessive levels of pro-inflammatory cytokines (29, 35).

##### Autonomic nervous system influence

The autonomic nervous system directly connects the brain to peripheral organs and tissues. Its two separate branches send opposing messages, sympathetic arousal and parasympathetic relaxation.

Sympathetic innervation links the brain directly to the adrenal medulla and readies the body for “fight-or-flight.” Upon sympathetic activation, the adrenal medulla releases catecholamines, epinephrine, and norepinephrine, that increase heart rate, blood pressure, and breathing rate and diverts blood from nonessential organs to the major muscle groups and the brain. Although catecholamines have short half-lives and metabolized quickly in the blood, the SNS also directly innervates secondary lymphoid structures that act as immune cell reservoirs. Therefore, chronic sympathetic activation and release of norepinephrine can lead to immune dysregulation (3). For example, norepinephrine promotes nuclear factor-kappa B (NF-κB) activation (36), which increases gene expression of several pro-inflammatory mediators, in turn enhancing inflammation (37, 38). In addition, epinephrine increases IL-6 and TNF-α production during stress (39). Thus, epinephrine and norepinephrine can induce pro-inflammatory cytokine production and enhance systemic inflammation.

The parasympathetic nervous system (PNS) opposes the sympathetic nervous system in a variety of ways such as slowing heart rate, decreasing breathing rate, increasing digestion, and calming mood. The vagus nerve has afferent and efferent nerve fibers for bi-directional communication between the brain and periphery (40). Parasympathetic activation causes acetylcholine release (41). Acetylcholine can bind to the α7 nicotinic acetylcholinergic receptor (nAChR) on an immune cell's surface (42). α7 nAChR stimulation inhibits NF-κB from altering inflammatory expression; resulting in decreased cytokine production and anti-inflammatory effects (43, 44). The PNS controls organs and bodily functions during rest; thus, in a chronically stressed individual, the PNS' reset on immune function dissipates as the “switch” to sympathetic dominance occurs (45).

The maintenance of a well-balanced autonomic nervous system, meaning vagal dominance during times of rest and the dynamic, variable activation of the PNS, has been linked to the emotional reactivity and stress vulnerability (46, 47). Porges' Polyvagal Theory utilizes an evolutionary and developmental approach to linking PNS activity and social communication with a hierarchy of circuits that support adaptive response to restful, potentially dangerous, and life-threatening environments (46). In what might appear as contradiction, Thayer and Lane proposed the Neurovisceral Integration model to describe how stressors (regardless of source) converge on the brain from the peripheral sensations, the messages are integrated, and the flexibility of the PNS to respond is critical to understanding how the individual's physiology is regulated (47). Despite which lens is used, heart rate variability (HRV) estimates the influence of PNS over the SNS and greater variability in PNS activation (i.e., a more well-balanced and flexible body) results in higher HRV. Thus, individuals with higher HRV function better and have greater well-being (e.g., socially, emotionally, mentally, physiologically), including lower inflammation, than those with lower HRV (45). For primary care clinicians and healthcare providers working with vulnerable populations, HRV may serve as a good estimate of how well the individual is functioning (48), including an indirect proxy for immune function.

Although this review focuses primarily on the intersection between neuroendocrine and immune systems, we recognize there are additional pathways that lead to resolution of inflammation (49–52). For example, lipid based mediators such as resolvins, protectins, and maresins play a critical role in shutting down and clearing the acute inflammatory response (53). Furthermore, these bioactive lipids appear to block NF-κB activation (50), mirroring the effects of cortisol and acetylcholine. For a more thorough evaluation of these pathways, see Dalli and Serhan's (49) and Chiurchiu and colleagues' (54) recent reviews.

### Conclusions

Acute inflammation in response to injury or infection is adaptive and successfully supports the careful orchestration of both the innate and adaptive immune response. However, constant or repetitive activation of the immune system whether psychologically or organically (i.e., antigen, injury) related leads to long-term exposure resulting in low grade inflammation. This chronic inflammation disrupts multiple systems due its effect on the nervous system as well as locally via cytokine receptor expression throughout multiple bodily tissues.

As an *integrated system*, the body requires a *universal way* of communicating between its distinct anatomical parts, i.e., a common physiological mechanism. Inflammation, regulated by pro- and anti-inflammatory cytokine production, may be the key to understanding how disease develops and progresses within the body. Hence, the prevailing *siloed view of diseases being independent of each other* and therefore needing to be managed by discrete specific interventions is no longer tenable, and as experience shows has largely limited success. The “phenotypic” characteristics of a disease is just one *representation of the disruptions in the whole system disruption*. In integrated systems, the disturbance of one variable “causes” effects, cascading via multiple pathways, on other key factors and invariably is associated with feedback to modulate the *behavior* of the “causative variable.”

For example, external and internal factors can result in the same phenotypical outcome; low socioeconomic status and excessive adipose tissue both are linked to chronic inflammation. This elevated inflammation leads to increased brain cytokine signaling and impairs learning and memory and supports depressive symptoms or *sickness behavior*; this feedback results in further propagation of the negative or unhealthy systemic cycle. Thus, uncontrolled or dysregulated inflammation unites the manifestation of chronic physical and mental diseases that often are prevalent in high-stress, vulnerable populations (e.g., ethnic minority, lower socioeconomic status, etc.).

## The role of inflammation in common conditions

Currently the etiology of most diseases is understood in terms of causative external factors and/or altered internal biomedical changes resulting in a disease's macroscopic and/or microscopic appearances. The obvious but not often raised question though should be: *how are these changes facilitated*?

Most disease—acute and chronic—results from inflammation. Dysregulation of the neuroendocrine-immune balance, regardless of being stimulated by external factors like “stress” and invasive pathogens or internal factors like metabolic derangements like diabetes or renal failure, leads to uncontrolled immune cell activity. The concurrent loss of anti-inflammatory mechanisms results in elevated cytokine loads that in turn activate NF-κB and gene transcription regulation (33, 37, 55, 56).

In addition, it has become clear that many diseases are linked through the diseasome^1^ (57, 58), indicating that the genome activity triggers common pathways of “related” diseases, like heart disease and diabetes. Disease classifications provide a *phenotypic* naming without regard to underlying gene and physiological network interactions. These insights challenge current approaches to “multimorbidity”—the collection of phenotypic diseases in the one person is the emergent outcome of finely tuned interconnected physiological network responses (59).

Here we see one of nature's principles at work—*system design* and *efficiency*. The finely tuned balance between pro- and anti-inflammatory activities provides the blueprint to effectively and efficiently respond to all forms of internal and external disturbance that threatens the organism's viability.

We now outline some of the significant inflammatory mechanisms behind many common chronic diseases in greater, but by no means comprehensive, detail. In particular, we highlight the connectedness of inflammatory activation through stimulation of the hypothalamic-pituitary-adrenal (HPA) axis and chronic sympathetic activation on the promotion of disease.

### Atopy, asthma, and allergic rhinoconjunctivitis

Allergic airway diseases (AAD, asthma and allergic rhinitis) have risen in prevalence over the last 30 years; asthma affects about 8% and allergic rhinitis between 15 and 20% of the population (60). Allergy refers to the inappropriately damaging response to ubiquitous and intrinsically relatively benign agents in the environment—perennial triggers include house dust mite, animal danders, molds and cockroach proteins, seasonal triggers involve pollens from grasses, trees, and weeds.

**Figure 4:**
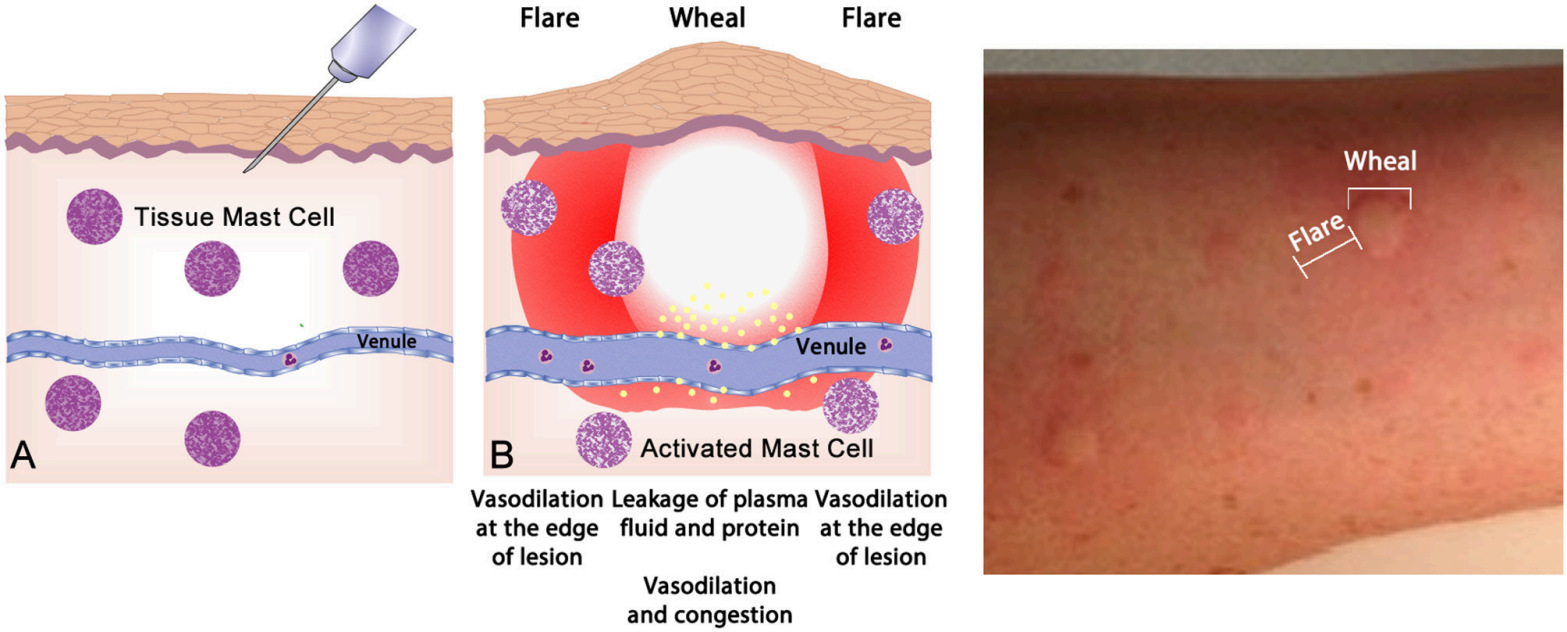
The wheal-and-flare reaction in the skin. **(A)** Normal basal or a non-reactive state for mast cells in tissue. The needle injects a known allergic antigen or allergen. **(B)** Antigens provoking allergic reactions drive specific-IgE production by plasma cells, assisted by TH2 cells. Surface bound IgE on mast cells is cross-linked by secondary exposure to allergen, causing release of preformed (e.g., histamine) and lipid-derived (e.g., leukotriene) mediators which drive the vascular events of allergy (vasodilatation and vascular fluid leak) manifesting as a “wheal and flare.” This photograph depicts a typical wheal-and-flare reaction in the skin in response allergen injection.

Up to one half of the population display *atopy*, an inherited tendency toward excessive production of IgE antibodies to ubiquitous antigens driven by T_H_2 type T cells (61). Atopy is caused by allergen-specific IgE reaction and proven by epicutaneous skin testing (“skin prick testing”—Figure 4) or measurement of blood allergen-specific IgE^2^. Such tests reveal the presence of sensitization, but only a subset of such individuals will go on to develop clinically apparent allergic disease—allergic dermatitis (eczema), allergic rhinitis, asthma, food allergy, and anaphylaxis. Atopy has a strong genetic determination; having one atopy parent confers around a 50%, whilst having two atopic parents will display a 70% likelihood of manifesting allergic disease (62).

Intermittent reversible airways obstruction, chronic (often eosinophilic) bronchitis, and bronchial smooth muscle hypertrophy with hypersensitivity to bronchoconstrictors defines the clinicopathological triad of asthma. It results from repeated episodes of airway-based immediate and late-phase allergic reactions mediated by leucotrienes (LTC4, LTD4, LTE4)—all potent mast-cell-derived bronchoconstrictors. However, 30% of patients do not have an identifiable allergic trigger and may result from hyperresponsiveness to various nonspecific stimuli such as intercurrent infections, drugs, colds, esophageal reflux, and exercise. Of these so-called “non-allergic” asthmatics, there are a number of sub-phenotypes, with some displaying “Type 2” responses (IL4- and IL13- dominated) *without* IgE-production, and with some subsets displaying T_H_17/neutrophilic dominance, and others marked by excessive uncontrolled T_H_1 activity (63).

**Figure 5:**
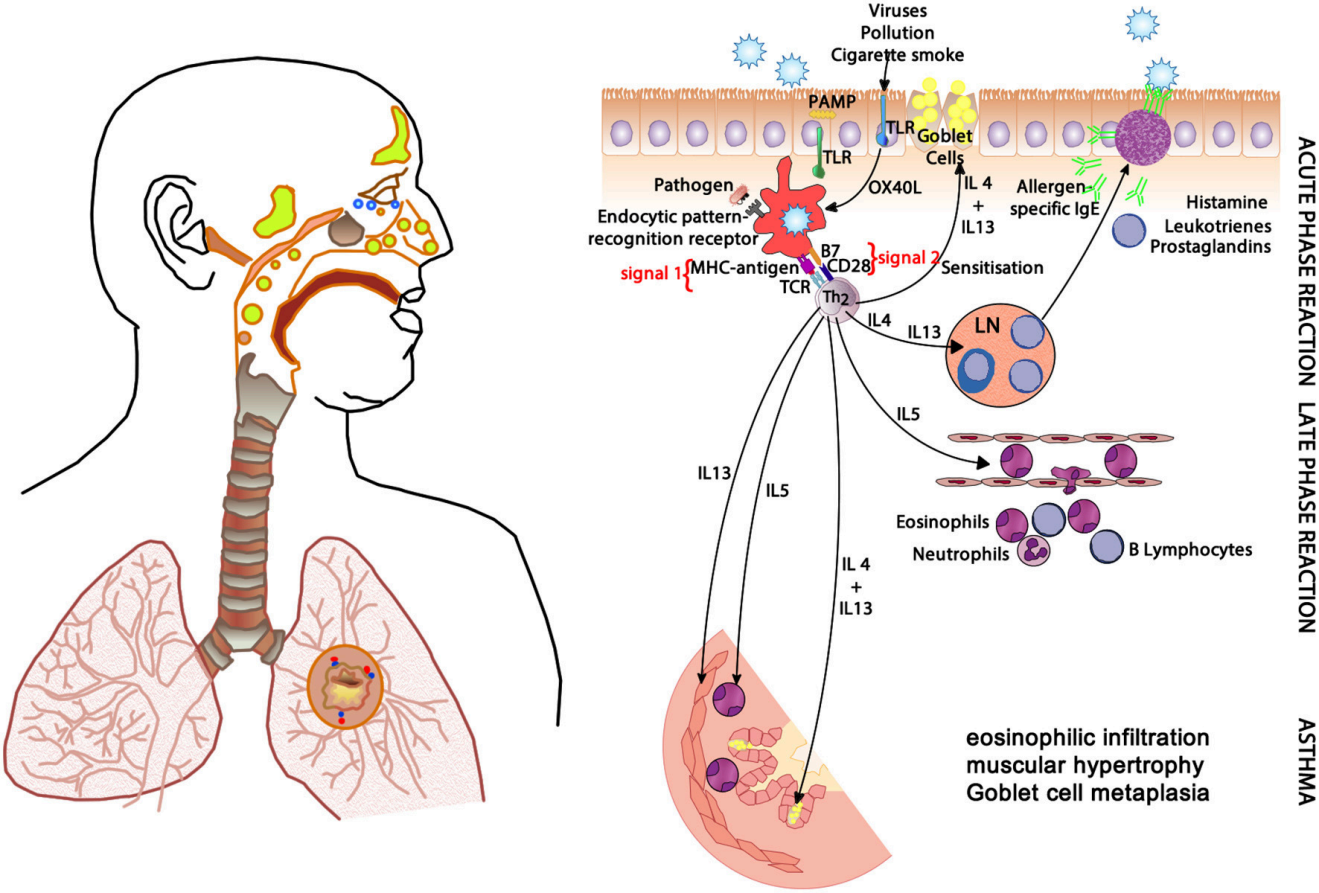
The clinical changes and physiological mechanisms of allergic respiratory disease. Antigen-presenting cells (APCs) present allergen-derived peptides lodged within the MHC molecule to T cells within a T_H_2 milieu, resulting in allergy mediated by eosinophils and IgE derived from plasma cells (transformed from activated B cells). PAMP, pathogen-associated molecular pattern; TLR, toll-like receptor; MHC, major histocompatibility complex; TCR, T cell receptor; IgE, immunoglobulin E; OX40L, CD252; B7, CD28; IL-4, interleukin-4; IL-5, interleukin-5; IL-13, interleukin-13.

**Figure 6:**
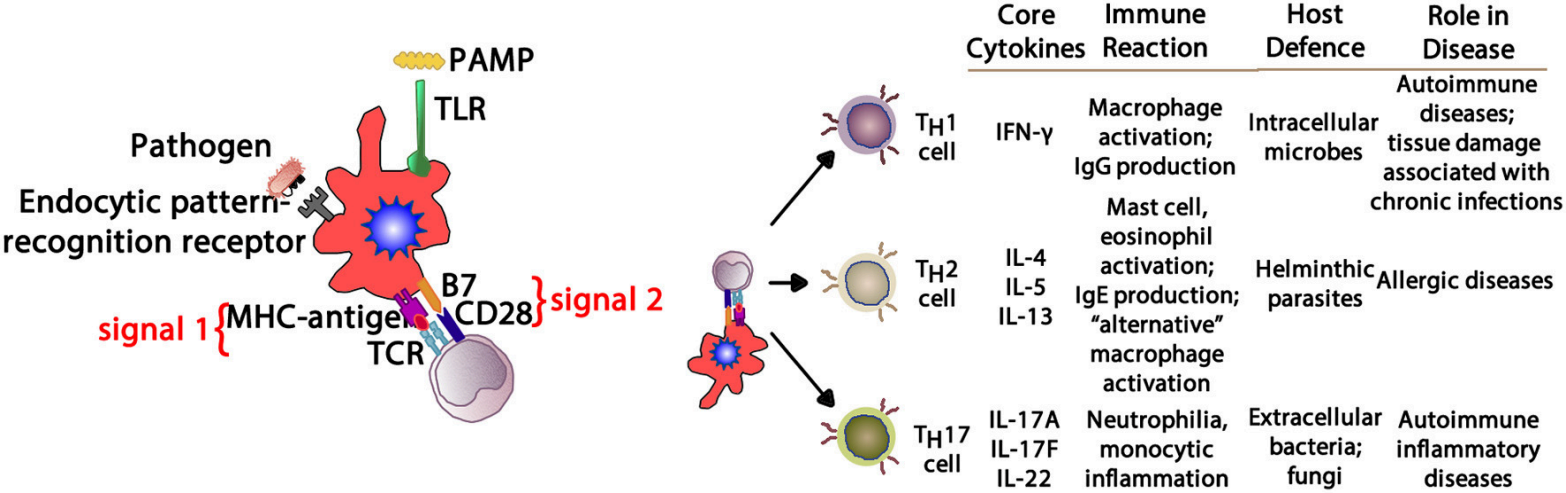
Depending upon the cytokine milieu present at the time of antigen presentation (signal 1) and costimulation (signal 2), three T cell polarization pathways can develop. IFN-γ supports T_H_1 deviation that mediates macrophage-mediated reactions. IL-4, IL-5, and IL-13 create a T_H_2 environment that drives eosinophil and IgE production. IL-17A, IL-17F, and IL-22 provide aT_H_17 environment driving neutrophilic and some autoimmune phenomena. PAMP, pathogen-associated molecular pattern; TLR, toll-like receptor; MHC, major histocompatibility complex; TCR, T cell receptor; IFN- γ, interferon-gamma; IgG, immunoglobulin G; IgE, immunoglobulin E; IL-4, interleukin-4; IL-5, interleukin-5; IL-13, interleukin-13; IL-17A, interleukin-17A; IL-17F, interleukin-17F; IL-22, interleukin-22.

In allergic respiratory disease, antigens are ingested, processed and presented by antigen-presenting cells (APCs—the prototype is the dendritic cell) (64), which present proteolytically-derived components of the allergen in the cleft of a cell-surface-bound MHC (HLA) molecule to passing T cells in the context of a specific cytokine milieu (Figure 5) (65). When T cells with TCRs displaying specificity for the MHC:peptide complex bind to form the trimolecular complex (MHC:peptide:TCR—“signal 1”), T cell activation is possible. However, in order for T cell activation to occur, another signal must also be delivered to the APC for “co-stimulation” to occur (B7:CD28 is a classic example of “signal 2”), and is induced after the APC recognizes invariant signals including pathogen-associated molecular patterns (PAMPs) within the inciting antigen which flag the protein as foreign and potentially dangerous (66). The cytokine milieu existing at the time of antigen presentation dictates the T cell pathway that is followed (Figure 6): if host and environmental factors result in a dominance of IL-4 at the time of antigen presentation, T cells adopt a “T_H_2” polarization and drive allergic (and anti-parasitic) activities (67).

### Autoimmunity

**Figure 7:**
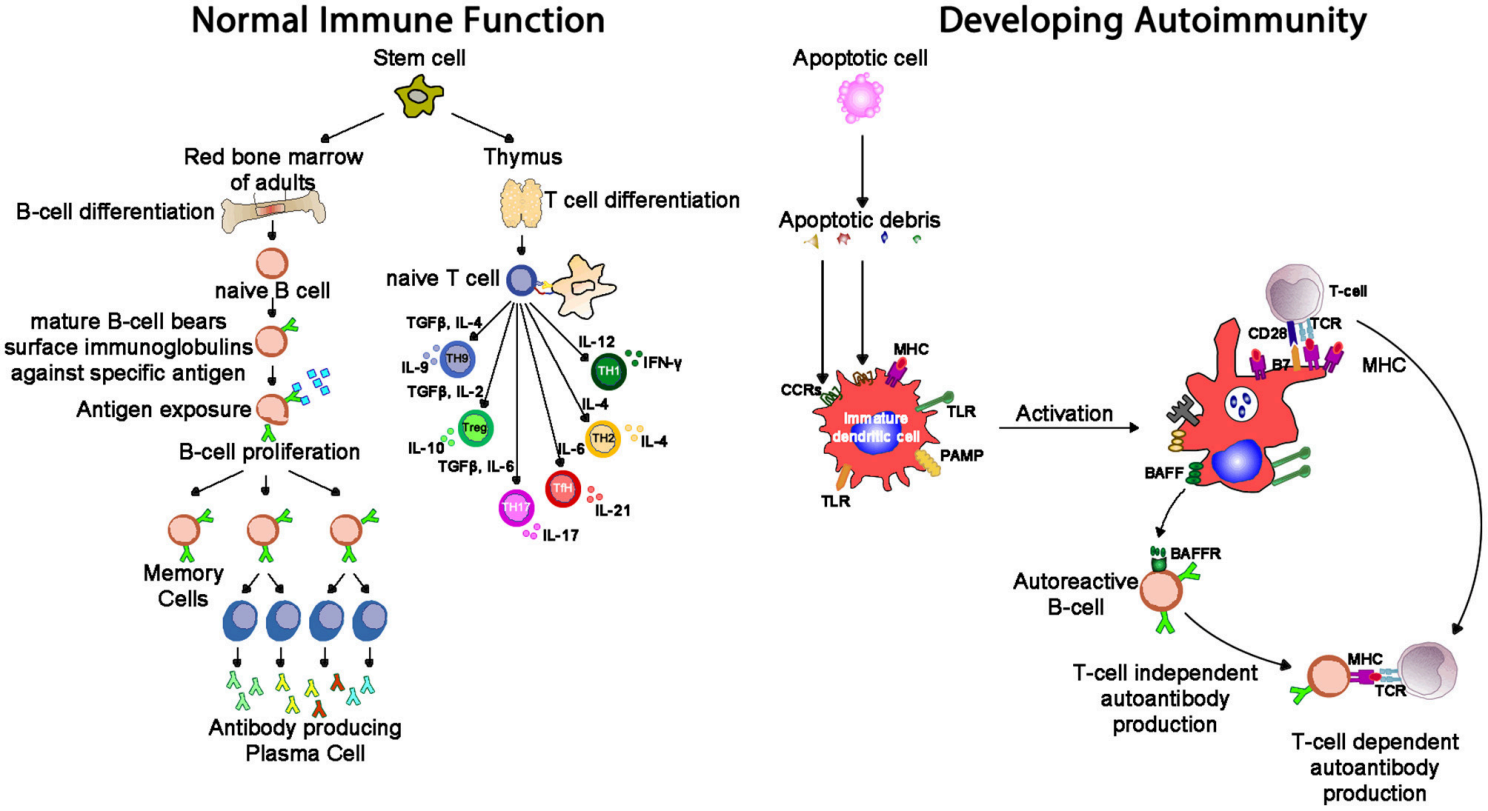
Whereas, normal immunity is marked by tolerance to self-structures (mediated by central and peripheral regulatory pathways), autoimmunity results from aberrant activation of autoreactive T and B cells, responsible for inflammation seen in autoimmune diseases. One mechanism of tolerance breakdown shown in the figure involves impaired clearance of apoptotic debris. IL-2, interleukin-2; IL-4, interleukin-4; IL-6, interleukin-6; IL-9, interleukin-9; IL-10, interleukin-10; IL-17, interleukin-17; TGF-β, transforming growth factor-beta; PAMP, pathogen-associated molecular pattern; TLR, toll-like receptor; MHC, major histocompatibility complex; TCR, T cell receptor; CCR, C-C chemokine receptors; BAFF, B cell activating factor; BAFF-R, B cell activating factor receptor.

Autoimmune conditions affect around 5% of the population (68), and manifest with a range of systemic (e.g., lupus, scleroderma) and organ-specific (e.g., chronic urticaria, thyroiditis) conditions. Lymphocytes develop through random rearrangement of their genetic segments, generating a huge diversity of recognition through T cell and B cell receptors which then, upon binding specific antigens in the correct conditions, drive cellular activation, and proliferation (69). This random process of recombination runs the risk of generating self-reactive lymphocytes. While most of these self-reactive lymphocytes are deleted in an apoptotic (programmed cell death) process producing self-tolerance (70), a small proportion of lymphocytes escape this process of thymic (T cell) and marrow (B cell) central tolerance: this results in a potential capacity for autoimmune pathology (Figure 7). The risk of autoimmunity is minimized by an additional layer of peripheral tolerance mechanisms which prevent activation of autoimmune cells by self-antigens; the most critical of these mechanisms is the need for a second signal (signal 2), also known as “co-stimulation” before T cell activation occurs (71). This second signal, as discussed above, is induced by recognition of “foreignness” or “danger” markers on the antigen like pathogen associated molecular patterns (PAMPs) or damage associated molecular patterns [DAMPs, (72)].

When both processes of central and peripheral tolerance fail, autoimmune disease is the result. Autoimmune disease must be distinguished from autoimmune potential (or “autoimmunity”), which creates a potential for self-antigen-triggered inflammation, but requiring additional triggers for the process to be initiated. Autoantibodies (e.g., ANA) and autoimmune cells can be present in a healthy individual without autoimmune disease being present. It has been suggested that autoantibodies may play an adaptive role in “mopping up” the products of tissue destruction and, equally, autoimmune T cells may assist with adaptive immune regulation and tolerance development through release of regulatory or immunosuppressive cytokines.

Developing T cells leave the bone marrow after maturing from stem cells and migrating to the thymus—here, cells with high reactivity to self-structures are deleted through apoptosis (73). Transiently, varied antigens from a host of tissues (e.g., testis, adrenal, pancreas) are expressed ectopically in the thymus in order to facilitate this central tolerance process: a monogeneic disorder where this machinery fails (AIRE mutation) manifests with multiple autoimmune endocrinopathies [e.g., diabetes type-I, hypoparathyroidism, and hypoadrenalism, (74)]. While some self-reactive T cells leave the thymus intact, peripheral tolerance mechanisms (e.g., T-regulator cell activity, anergy, apoptosis) further reduce autoimmune risk activity (75). Another monogeneic disorder, IPEX (Immune dysregulation polyendocrinopathy enteropathy X-linked syndrome) stops the generation of T_REG_ cells (mentioned above), resulting in generalized autoimmune disease that especially affects the endocrine organs (e.g., thyroid, pancreatic islet cells, gonads). Similarly, regulatory pathways lead to B cell development of central and peripheral tolerance (76).

The detection of antibodies in the serum of individuals in whom a diagnosis of autoimmune disease is suspected confers great diagnostic assistance in many conditions—examples include the detection of ANA in systemic lupus erythematosus (SLE), anti-cyclic citrullinated peptide (anti-CCP) in rheumatoid arthritis, and tissue transglutaminase (tTG) antibodies in coeliac. However, it is important not to extrapolate from the presence of these antibodies to the conclusion that they are pathogenic. Indeed, the majority of autoimmune conditions in which antibodies can be identified are in fact mediated dominantly by T cell mediated pathology, with the antibodies representing little more than diagnostically useful epiphenomena. Examples of these cell-mediated conditions, which are often dominated by excessive activity of the relatively newly-defined T_H_17 (IL-17 secreting) pathway and involve lymphocytic and/or neutrophilic infiltration and inflammatory tissue destruction, include type-1 diabetes, psoriasis, Hashimoto's thyroiditis, multiple sclerosis, rheumatoid arthritis, and Crohn's disease. In contrast, there are many well-known conditions for which autoantibody-related pathogenesis has been proven (by such natural experiments as neonatal disease induction through transplacental antibody passage): these include myasthenia gravis (acetylcholine receptor antibodies), Grave's disease (TSH-receptor stimulating antibodies), and SSA(Ro)-mediated neonatal lupus and congenital heart block.

Autoimmune conditions represent the end result of autoantibody-driven pathology or tissue inflammation and damage by self-reactive lymphocytes (77, 78). Autoimmune pathology results from a combination of genetic and environmental factors and is often chronic, progressive and self-perpetuating (79), although spontaneous remission is also possible in some cases where organ regeneration can occur (e.g., autoimmune hepatitis). Factors impacting upon the risk of autoimmunity include defective tolerance or regulation [e.g., genetically-determined defects of normal apoptosis or T_REG_ cell development (80)]; abnormal display of self-antigens (e.g., structural changes to self-proteins induced by cellular stress or injury); and excessive initial inflammation and innate immune activation (81) (e.g., infections or cell injury providing PAMPs or DAMPs that trigger expression of signal 2, providing co-stimulation to facilitate autoimmune responses).

Host genetic determinants of autoimmune risk cluster disproportionately around the MHC locus on chromosome 6, including HLA-DR (82): this is not surprising, given the central role of MHC-mediated antigen presentation in the normal processes of immune tolerogenesis and activation (83). However, partly as a result of genome-wide association studies in autoimmune populations, a number of non-HLA genetic risk determinants have also been identified: often, these genetic defects affect levels or function of cell surface receptors or cytokines with central roles in immune tolerance and activation (84), such as reduced IL-10 or increased IL-23 (a T_H_17-inducing cytokine).

There are, however, a proportion of individuals with these serological and cellular autoimmune phenomena who will, with further triggers, move on to develop overt autoimmune disease. The exact nature of this multistep process of autoimmune pathogenesis is still, however, only incompletely understood. Greater understanding of normal tolerance pathways and their maintenance (and loss) will provide growing insights into a range of immune pathologies, providing potential clues for prevention and modulation of autoimmune diseases, as well as management strategies in other settings, ranging from transplantation to malignancy, and even conception and pregnancy.

### Coronary artery disease

Cardiovascular disease, mostly involving coronary and cerebral arteries, is the world's largest cause of chronic morbidity and mortality. Its basis is atherosclerosis, a chronic inflammatory disease of blood vessels. The critical region of an atherosclerotic plague is its shoulder region which is infiltrated by activated T cells, macrophages and mast cells, which produce pro-inflammatory mediators and enzymes. Rupture of a plague triggers platelet aggregation and thrombus formation resulting in acute ischemia, clinical symptoms, and potential distal cell death (85, 86).

Macrophages are the hallmark of the atherosclerotic lesion. They import oxidized LDL (oxLDL) via scavenger and toll like receptors (TLR). Cholesterol is esterified in the cytoplasm and transforms macrophages into foam cells. Oxidized lipoproteins signal to the surrounding cells of the plaque, and especially so to the endothelium and foam cells, increasing the expression of adhesion molecules which in turn attract more monocytes and lymphocytes to the locally activated endothelium. Foam cells and activated endothelium produce pro-inflammatory cytokines—IL-1, IL-6, IL-12, IL-18, IFN-γ, and TNF-α–which exaggerate the inflammatory responses (85).

**Figure 8:**
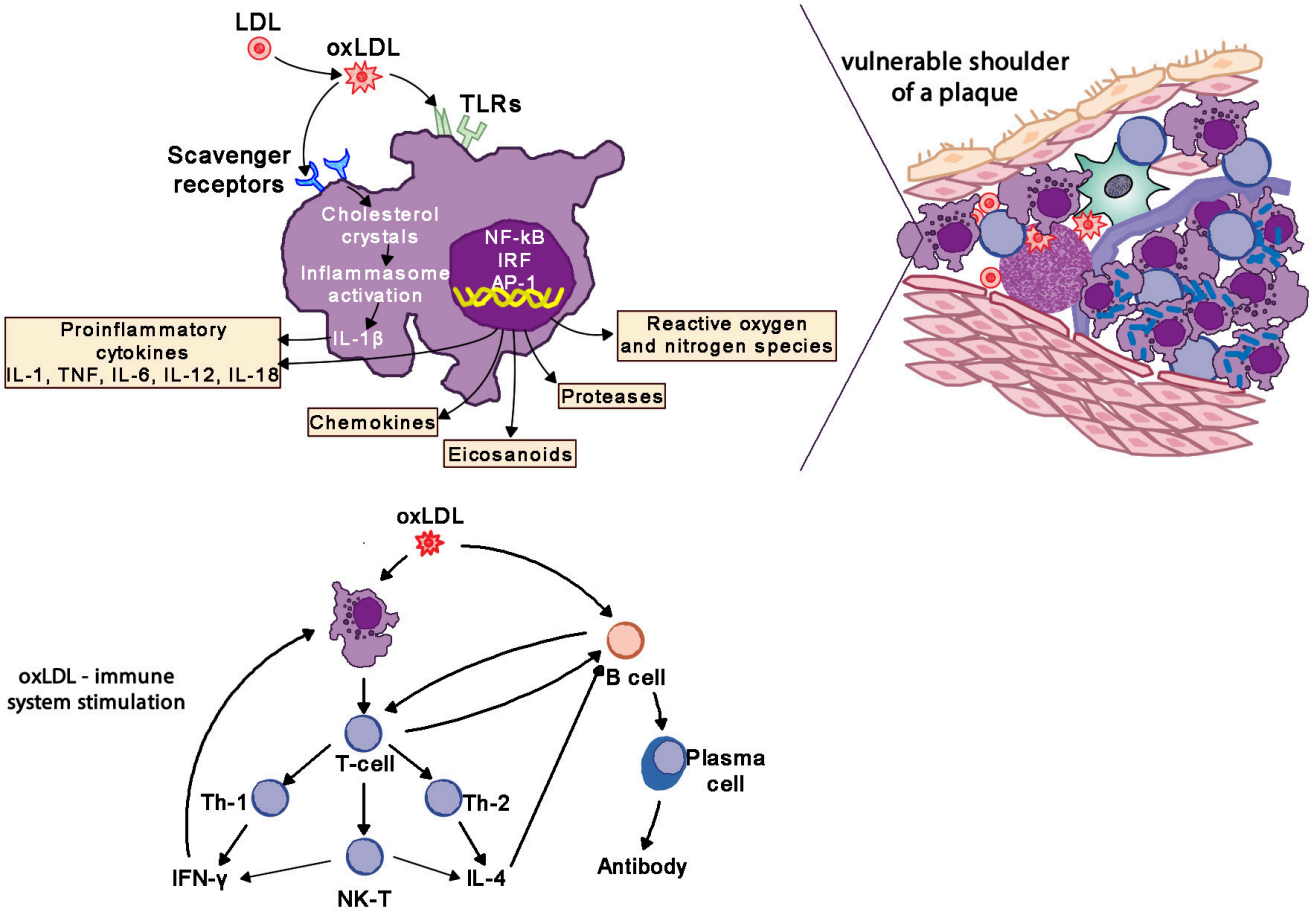
Oxidized LDL (oxLDL) activates pro-inflammatory pathways to drive inflammatory changes in coronary vascular disease. LDL, low-density liporptein; oxLDL, oxidized LDL; TLR, toll-like receptors; IL-1, interleukin-1; IL-6, interleukin-6; IL-12, interleukin-12; IL-18, interleukin-18; TNF, tumor necrosis factor; IL-4, interleukin-4; IFN-γ, interferon-gamma.

Atherosclerotic lesions contain macrophages, T cells, dendritic cells, proteoglycans and smooth muscle cells as well as cholesterol that infiltrates from the blood. Oxidized cholesterol triggers both innate and adaptive immune responses resulting in T cell activation and antibody production. T_H_2 cells produce IFN-γ, are pro-inflammatory and drive further inflammatory activity by activating macrophages, whereas T_H_2 cells produce IL-4 and IL-5. T_H_1 and T_H_2 activity is self-regulating with IFN-γ inhibiting T_H_2 cells and IL-4 inhibiting T_H_1 cells. Within the plaque T_H_1 activity is dominant but counterbalanced by IL-10 produced by macrophages and T_H_2 cells (Figure 8) (85, 86).

The interplay of the different immune cells has the potential to significantly influence the outcome of the inflammation in the plaque. In experimental studies the infusion of IFN-γ, IL-12, or IL-18 all increase atherosclerosis, whereas the infusion of antibodies to CD50L and oxLDL reduce atherosclerosis. TGF-β has been found to be anti-inflammatory, on the one hand limiting the recruitment of leukocytes, on the other promoting the synthesis of collagen and thus stabilization of the plaque (85, 86). In addition, organisms like chlamydia may activate TLRs and promote the inflammatory processes of atherosclerosis (4, 85, 87). Therapeutically statins inhibit the Rho/ROCK pathways and block the pro-inflammatory effects of IL-1β (86, 88).

### Cardiac arrhythmias

It is well established that abnormal cardiac autonomic regulation (cardiac autonomic remodeling—increased sympathetic coupled with decreased parasympathetic activity) enhances the risk for malignant cardiac arrhythmias and sudden cardiac death, particularly in patients with pre-existing cardiovascular disease (89). As was previously noted, sympathetic neural activation is pro-inflammatory (36) and parasympathetic regulation is anti-inflammatory (42), inflammation may play a central role in the genesis of cardiac rhythm disorders. Indeed, elevated levels of inflammatory biomarkers are associated with an increased risk for sudden cardiac death (90–94). For example, Hussein et al. (95), recently found that elevated levels of either interleukin-6 (IL-6) or C-reactive protein (CRP) were each associated with an increased risk for sudden cardiac death during a 17 year follow-up period in older individuals either with or without pre-existing cardiac disease (heart failure or incident myocardial infarction). The sudden cardiac death risk persisted for IL-6, but not CRP, even after adjustment for baseline risk factors (95).

Pro-inflammatory pathways may also play an important role in atrial fibrillation (AF) (94, 96, 97). Frustaci et al. (98) were the first to propose that inflammatory processes damage atria and promote electrical derangements that culminate in AF. Specially, they found lymphomononuclear infiltrates in atrial biopsies from patients with lone AF but never in control subjects (Wolf-Parkinson's-White syndrome patients). Subsequently, a number of clinical studies have established strong association between inflammatory biomarkers (CRP, IL-2, IL-6, IL-8, TNF-α; monocyte chemoattractant protein-1) and persistent and paroxysmal AF (94, 96, 97).

Given the strong association between inflammatory biomarkers and both AF and sudden cardiac death, disruption of pro-inflammatory or enhancement of anti-inflammatory pathways may prove to be important novel therapeutic targets for the management of cardiac arrhythmias. In fact, anti-inflammatory agents (e.g., non-steroidal anti-inflammatory drugs [NSAIDs] and corticosteroids) have been shown to reduce the risk for peri-operative AF (99). However, these agents increased rather than decreased the incidence of AF in other patient populations (99), while the effects of these agents on ventricular arrhythmias and sudden death remain to be determined.

### Obesity

**Figure 9:**
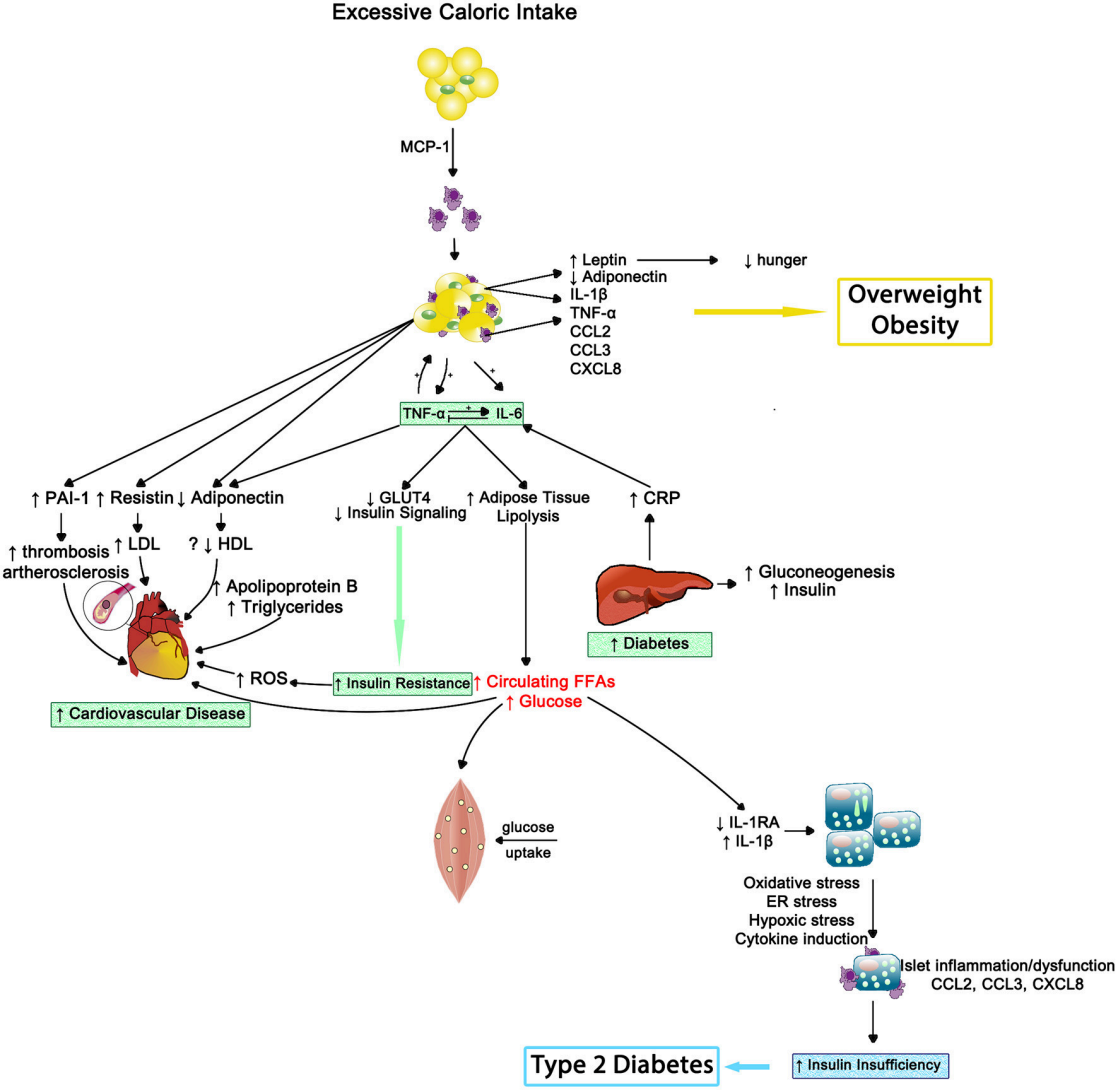
The inflammatory effects of obesity on insulin resistance, cardiovascular disease and type 2 diabetes—Inflammation results in impaired muscle cell metabolism and insulin insufficiency. MCP-1, monocyte chemotactic protein 1; IL-1β, interleukin-1beta; TNF-α, tumor necrosis factor alpha; IL-6, interleukin-6; CCL2, CC chemokine ligand 2; CCL3, CC chemokine ligand 3; CXCL8, CXC chemokine ligand 8; PAI1, plasminogen activator inhibitor type 1; LDL, low density lipoprotein; HDL, high density lipoprotein; ROS, reactive oxygen species; GLUT4, insulin-regulated glucose transporter type 4; CRP, C-reactive protein; FFAs, free fatty acids; IL-1RA, interleukin-1 receptor antagonist; ER, endoplasmic reticulum.

In 2013, the American Medical Association endorsed obesity as a disease and now it has surpassed tobacco smoking as the single most preventable cause of morbidity and premature mortality (100). Obesity can be estimated by body mass index (BMI), with 30–39 range indicating obese status and 40 or greater considered morbidly obese. Adipose tissue, especially centrally located, behaves much like an endocrine gland that can modulate other tissues' activities and be influenced by collocated nervous and immune systems (101, 102). As outlined in Figure 9, macrophages reside in adipose tissue. Whether a critical component of obesity's pathogenesis or due to the physical stress of carrying excess adipose tissue, obesity coincides with greater circulating pro-inflammatory cytokines; thus, obese individuals experience a state of chronic inflammation that appears to be dose-dependent and exists regardless of negative health *behavior*s and disease status (100, 103–105).

Stress appears to exacerbate the pro-inflammatory state in obese individuals. For example, obese women exhibited a greater inflammatory stress response than non-obese women to an acute stressor (106, 107). Pro-inflammatory cytokines such as IL-1, IL-6, and TNF-α produced by adipose tissue (108) or in response to stress can activate the hypothalamic-pituitary-adrenal (HPA) axis (109); hence the relationship among obesity, systemic inflammation, and stress reactivity is cyclical in nature. Prior to interrupting this cycle, clinicians and other health advocates must first be aware of its existence.

As expected, weight loss lowers inflammation including CRP, TNF-α, IL-6, and IL-18 with maintenance continuing for up to 2 years following diet alone or combined with physical activity intervention (110–112). Often combined (diet and physical activity) interventions are most effective due the reduction in fat mass composition; however, due to the intrinsic link between fat loss and increased physical activity, it is difficult to determine which factor is driving the reduction in inflammation. For instance, more physical activity resulted in lower chronic inflammation compared to less physical activity, but when BMI and leptin levels were accounted for the relationship no longer existed (113). Furthermore, in a longitudinal study, increased low-grade inflammation was associated with greater adiposity, but not physical fitness (114). Taken together, despite the fact that physical activity and exercise are linked to lower inflammation, it appears that management of an individual's fat mass is a more critical factor to overall health because of its strong connection to elevated systemic inflammation.

### Insulin resistance and type-2 diabetes

Insulin resistance occurs when an individual's cells become insensitive to insulin's message to absorb glucose from the bloodstream. This insensitivity results in elevated production of insulin by the pancreas; starting a never ending negative cycle that often results in beta-cell fatigue or apoptosis and the development of metabolic diseases such as type-2 diabetes (115). Systemic inflammation appears to play a pivotal role in both metabolic abnormalities (see Figure 9).

Higher CRP levels have been related to insulin resistance, suggesting that elevated inflammation may drive progression of type 2 diabetes (116). Indeed, elevated CRP and IL-6 independently predicted the development of type-2 diabetes across a 4-year period in the Women's Health Study, after controlling for BMI and family history of type-2 diabetes (117). Hence, systemic inflammation alone can promote insulin resistance, but obesity appears to compound the situation.

As noted in the obesity section, increased BMI or obesity is related to greater systemic inflammation (e.g., CRP, IL-6, and TNF-α levels). Obesity-induced inflammation has also been linked to the development of insulin resistance, even when controlling for BMI (118, 119), indicating the additional physical stress of being insulin resistant on increasing chronic inflammation beyond fat mass. Furthermore, insulin resistance exacerbates inflammatory-related diseases such as hepatitis-C infection (120) whereas administration of TNF-α inhibitors in patients with rheumatoid arthritis increased their insulin sensitivity (121).

The evidence linking inflammation and insulin resistance and/or type-2 diabetes is strong (122). Fetuin-A, a liver acute phase protein, may provide the key mechanism. Elevated fetuin-A levels are associated with cross-sectional occurrence and the longitudinal development of insulin resistance and/or type-2 diabetes (123, 124). Fetuin-A has two endogenous effects that support the data; 1. it inhibits the insulin-stimulated insulin receptor tyrosine kinase—reducing insulin sensitivity (125, 126)—and 2. it activates toll-like receptor 4 (TLR4) in adipose tissue—increasing inflammation (127). Thus, fetuin-A could be novel target.

Finally, *behavioral* and pharmaceutical interventions used to treat type-2 diabetes cause reductions in inflammation. Similar to BMI reductions via lifestyle management leading to lower inflammation, a weight loss intervention decreased plasma IL-18 and increased insulin sensitivity (111); suggesting that efforts to reduce BMI and/or adipose tissue can improve sensitivity to insulin. Glycemic control medications like metformin and rosiglitazone concurrently reduce inflammation and increase insulin sensitivity (128, 129); yet, the exact mechanism underlying the inflammatory improvements remain elusive.

### Depression

Mental and physical fatigue are primary symptoms of depression that include lack of focus, little or no motivation, lack of interest in previously enjoyed activities, sleep and appetite disturbances, irritability, hopelessness, and social isolation (130). Dysregulation of the *HPA-axis* has been associated with depression for several decades (24, 131); however, the relationship between depression and inflammation has evolved more recently (132, 133).

**Figure 10:**
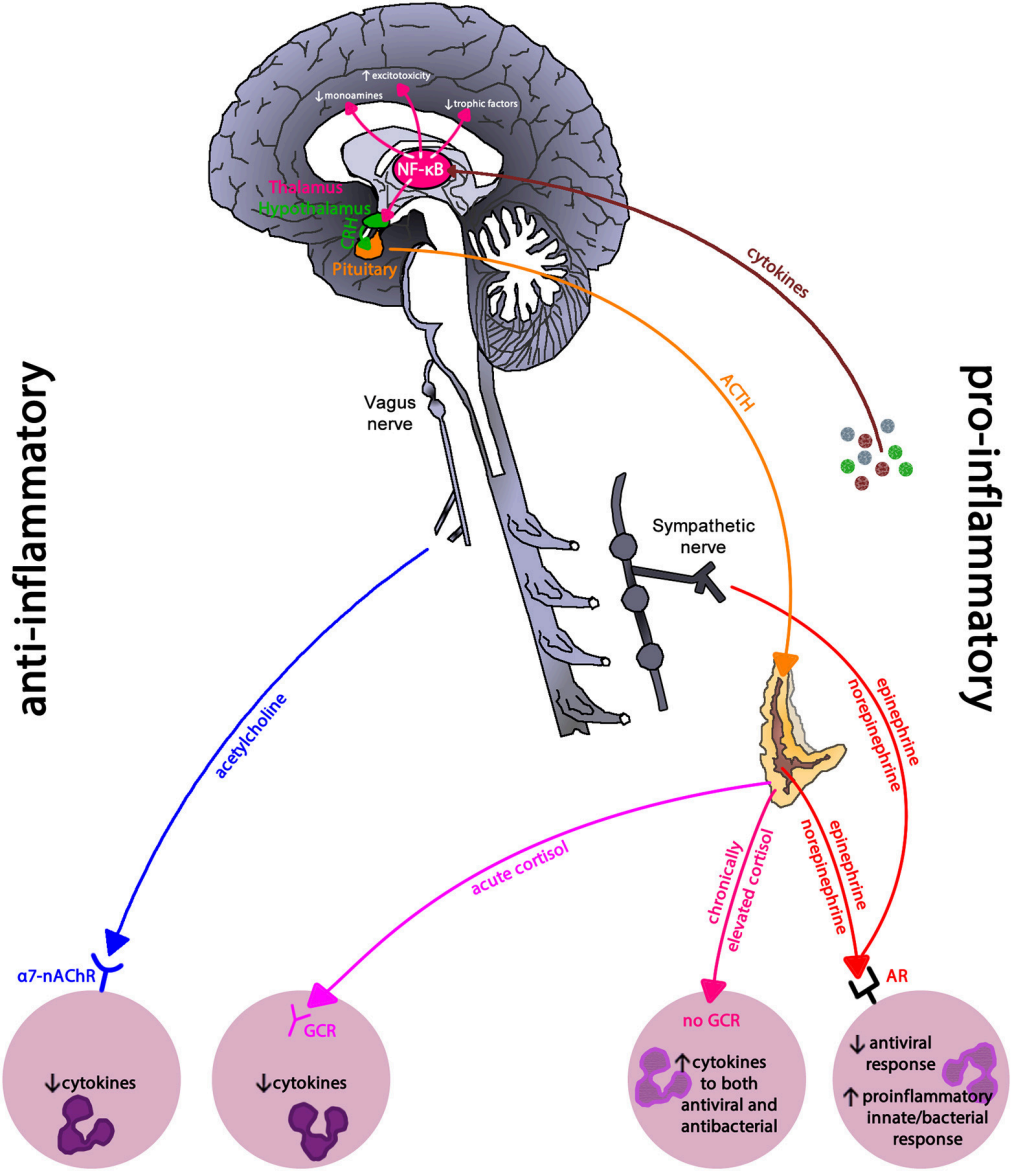
Inflammatory regulation in depression. Inflammatory pathways use the pro-inflammatory NF-κB signaling pathway to drive the neurohormonal changes seen in depression. Reduced glucocorticoid receptor expression and parasympathetic activity leaves immune cells in a primed pro-inflammatory state due to sympathetic nervous system dominance. NF-κB, nuclear-factor kappa-B; CRH, corticoptropin-releasing hormone; ACTH, adrenocorticotropin hormone; a7-nAChR, alpha-7 nicotinic acetylcholine receptor; GCR, glucocorticoid receptor; AR, adrenergic receptor. This figure is an modified/updated version of a previously published figure by Bennett and Sturmberg (144).

Depression is often co-morbid with inflammatory-related diseases such as cardiovascular disease and cancer (134, 135); suggesting that chronic inflammation may be linked to depression. Indeed, Major Depressive Disorder and depressive symptoms among community dwelling adults are linked to chronic inflammation, and severity showing a dose dependent pattern (136–138). Uncontrolled or dysregulated immune cells, due to prolonged and exaggerated stress activation, may drive this observed relationship (see Figure 10). Compared to immune cells from non-depressed individuals, immune cells from those with depression have greater NF-κB activity due to reduced cortisol sensitivity (139, 140), creating an intracellular environment primed toward sustained pro-inflammatory cytokine production.

The relationship between depression and inflammation appears bidirectional. For example, *sickness behavior* or depression-like symptoms including negative mood, fatigue, and psychomotor slowing in otherwise healthy volunteers can be created via exposure to pro-inflammatory cytokines (1, 141, 142). Cytokines influence the production and metabolism of neurotransmitters such as serotonin and dopamine, which play critical roles in mood (143). Furthermore, the antidepressant effect of serotonin and norepinephrine reuptake inhibitors in clinically depressed individuals increases when administered with non-steroidal anti-inflammatories like COX-2 inhibitors or aspirin compared to those who receive the antidepressant and a placebo (133).

Depression especially major depressive disorder can be cyclical in nature; therefore, it is difficult to determine the originating event: increased systemic inflammation or increased depressive symptomology (144). Does inflammation matter to mental health? In light of the substantial depression-inflammation link, the answer is yes (145).

Moreover, other mental health disorders such as schizophrenia, bipolar disorder, and post-traumatic stress disorders are also linked to elevated inflammation (146, 147). Thus, clinicians need to be acutely aware of this mental-physical relationship when treating a patient with chronic inflammatory disease or one with depression and potentially other neuropsychiatric conditions. Chronic inflammation whether a driver or result of mental disorders begins to blur the line between our understanding of physical and mental health conditions as unique or independent experiences on the individual.

### Osteoarthritis

Cartilage destruction, subchondral bone remodeling and inflammation of the synovial membrane are the classical features of osteoarthritis. Initially osteoarthritis was thought of as a “wear and tear” disease leading to loss of cartilage, clinically the main feature; however, it is now clear that synovitis is the main driver of the disease. Epidemiological studies have shown that the progression of osteoarthritis correlates with the amount of pro-inflammatory cytokines, in particular IL-1β, TNF-α, and IL-6, in synovial fluid (148–150).

Synovial membrane inflammation, characterized by synovial lining hyperplasia, infiltration of macrophages, and lymphocytes, neo-angiogenesis, and fibrosis, has emerged as an important and early feature in osteoarthritis. Synovitis contributes to the increase in the pro-inflammatory cytokines IL-1β and TNF-α in synovial fluid, synovial membrane, subchondral bone, and cartilage (150–152). The degree of synovitis clinically correlates with pain and loss of function, osteophyte formation and progression of cartilage loss (152). Inflammation can be triggered by infectious and non-infectious stimuli like hyaluronic acid, proteins in synovial fluid and crystals resulting in activation of pattern-recognition receptors (PRRs) and toll-like receptors (TLRs) increasing synovial fluid inflammatory cytokines like IL-1β and IL-17 (149).

IL-1β and TNF-α, as well as the stimulation of PRRs and TLRs, shift the otherwise tightly controlled anabolic/catabolic cartilage homeostasis within the chondrocytes toward a more catabolic state. The increased production of nitrous oxide, radical oxygen species and prostaglandin E2 increase the release of matrix metalloproteinases (MMPs) and inhibit the expression of type-II collagen and proteoglycans in chondrocytes resulting in cartilage destruction (148–152).

**Figure 11:**
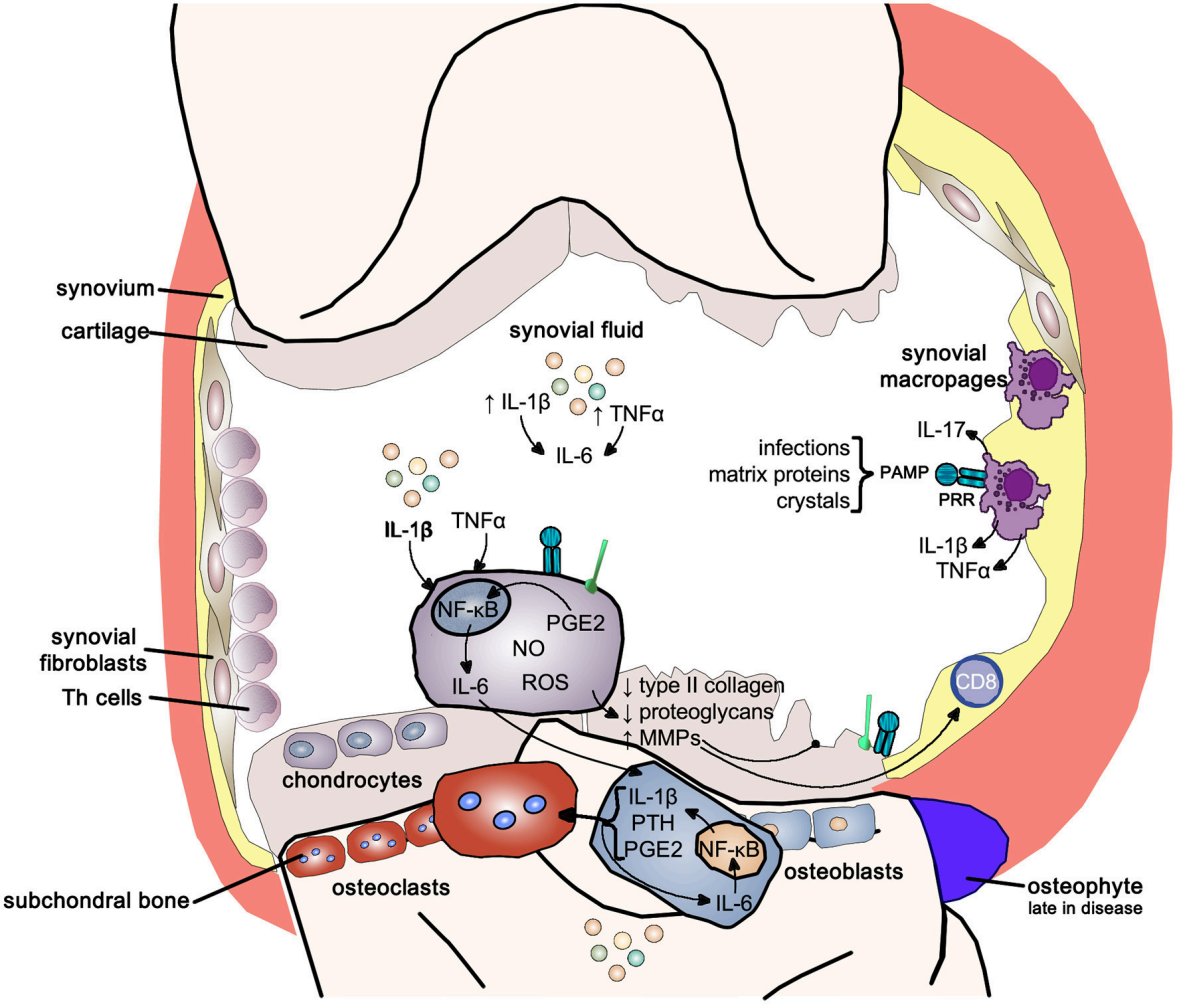
Inflammatory pathways in osteoarthritis. Inflammatory pathways use the pro-inflammatory NF-κB signaling pathway to drive the joint changes seen in osteoarthritis. NF-κB, nuclear-factor kappa-light-chain-enhancer of activated B cells; IL-1b, interleukin 1 beta; IL-6, interleukin-6; TNFα, tumor necrosis factor alpha; IL-17, interleukin-17; PAMP, pathogen-associated molecular pattern; PRR, pattern-recognition receptor; PGE2, prostaglandin E2; NO, nitric oxide; ROS, reactive oxygen species; MMPs, matrix metalloproteases; PTH, parathyroid hormone.

IL-1β and TNF-α also markedly upregulate the release of IL-6 from the chondrocyte, activating osteoblasts to release IL-1β, PTH and PGE2, which in turn activate osteoclast activity resulting in subchondral bone absorption. At the same time these mediators stimulate intercellular IL-6 production perpetuating osteoclast activity (148, 151). Osteophytes, reflecting new bone formation, emerge only late in the disease (Figure 11).

It is now recognized that osteoarthritis is a systemic disease and aging driven by low-grade inflammation and associated with common aging conditions like mental decline and cerebrovascular and cardiovascular disease (149). Obesity, and particularly visceral adipositas, result in a marked increase in pro-inflammatory cytokine production, and adipokines, rising blood sugar levels and ox-LDL all exacerbate low grade inflammatory activity (149). In particular adipokines have been related to directly disturbing cartilage homeostasis (150); the benefits of weight loss results more likely from decreased inflammatory load rather than reduced mechanical loading of joints.

### Aging

The role of inflammation is emerging as the most important mechanism of aging—referred to as inflammaging—and plays an integral role in most explanatory models (153–156). Aging is the consequence of the steady accumulation of cellular damage related to the failure of clearing damage-associated molecular patterns (DAMPs)—breakdown products of necrotic cells, extracellular ATP, uric acid, amyloid fibrils, free cholesterol crystals. DAMPs are detected by tissue innate immune cells—macrophages, microglia cells in the brain, Kupffer cells in the liver, osteoclasts in bone and mesangial cells in the kidney. The increasing load of DAMPs leads to the sustained activation of the inflammasome complex resulting in the release of pro-inflammatory cytokines (IL-6 and IL-18) causing ongoing low-grade chronic inflammation (154).

Chronic inflammation is characterized by the persistent low-level elevation of pro-inflammatory markers in the serum or in and around affected organ tissues, and is clinically associated with neurodegenerative diseases, metabolic disorders, cancer, musculoskeletal conditions, cardiovascular diseases, and frailty (154, 157). A 2-4-fold elevation of pro-inflammatory markers like IL-6, IL-18, TNF-α, and CRP and serum amyloid A has been found in people over 50 compared to younger ones (154, 158, 159). Of note, chronic low-grade inflammation in multiple organs can lead to functional decline even in the absence of specific diseases (154). In particular higher levels of IL-6 are associated with greater frailty and disability and clinically correlate with slow walking speed, poor muscle strength, poor lower leg function, and anemia (159). Amongst older patients those with frailty have higher pro-inflammatory and lower anti-inflammatory markers like cortisol and IL-10 (160).

**Figure 12:**
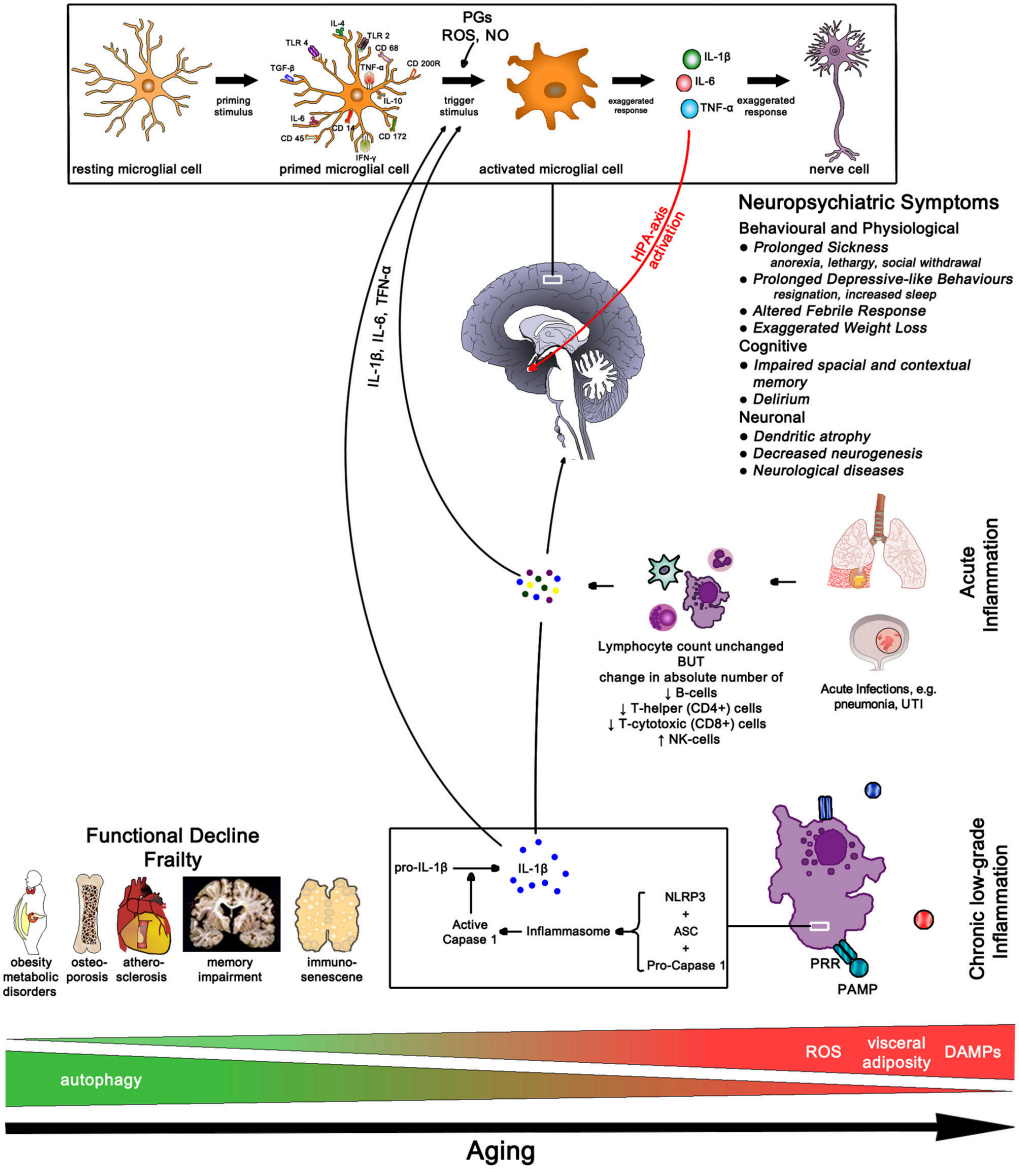
Aging Pathways—chronic low-grade inflammation results in functional decline and frailty. In older age the sudden rise in cytokine load due to an acute infection often results in neuropsychiatric conditions with slow and often incomplete recovery. IL-1β, interleukin-1 beta; IL-4, interleukin-4; IL-6, interleukin-6; IL-10, interleukin-10; TLR, toll-like receptor; TGF-β, transforming growth factor beta; TNF-α, tumor necrosis factor alpha; IFN-γ, interferon gamma; PGs, prostaglandins; ROS, radical oxygen species; NO, nitric oxide; NLRP3, nucleotide-binding domain leucine-rich repeat protein 3; ASC, apoptosis-associated speck-like protein containing a CARD; PRR, pattern recognition receptor; PAMP, pathogen-associated molecular pattern; DAMPs, damage-associated molecular patterns; UTI, urinary tract infection.

Immunosenescence describes the functional decline of the adaptive immune system with a hyperactivity of the innate immune system with aging. Whilst the absolute lymphocyte count remains unchanged, the absolute number of B-cells, T-helper (CD4+) and T-cytotoxic (CD8+) cells decreases but the number of NK-cells increases. There also is a decline of naïve T-cells—resulting in a decreased response to novel antigens, and the clonal expansion of T and B cells, especially those for herpesviruses like CMV, EBV, and VZV. Consequently, the aging body is less able to respond to new antigen presentation, and decline in memory response to known antigens, making the elderly more prone to infectious diseases (157, 161). In addition, the lifelong exposure to antigen causes progressive activation of innate immune cells further increasing pro-inflammatory cytokine release and chronic low-grade inflammation (42, 161) (Figure 12).

Two mechanisms for inflammaging have been proposed; one, mitochondrial dysfunction leading to a rise in radical oxygen species (ROS) which stimulate the formation of the inflammasome, and two, a decrease in autophagy function, i.e., more intracellular breakdown products become available to stimulate inflammasome formation (154). Fat plays an important role in immune function due to high levels of leucocyte infiltration—omental fat contributes 10–35% of basal circulating IL-6 (159)—thus perpetuating low-grade inflammation with increasing age-related weight gain which increases in particular the risk for diabetes in atherosclerosis (154). The passive diffusion of pro-inflammatory cytokines through the blood-brain barrier initially initiates an active anti-inflammatory response due to HPA-axis activation resulting in cortisol excretion from the adrenal gland. However, excessive pro-inflammatory load can activate CRP-releasing cells thus further activating the HPA-axis ultimately causing chronically elevated cortisol levels which in turn results in glucocorticoid insensitivity further perpetuating the pro-inflammatory state.

The higher peripheral inflammatory cytokine load affects the aging microglia cells which triggers an accelerated production of cytokines in the brain. The high brain cytokine load affects neuronal function resulting in the features of age-related mental function (162). Frailty, already a result of inflammaging due to high pro-inflammatory loads, is highly susceptible to exaggerated rise in pro-inflammatory loads resulting from infections, like urinary tract infection or pneumonia. The increase in IL-1β triggers maladaptive sickness *behavior*s affecting different parts of the brain; delirium results in working memory impairment and apathy (hippocampus), heightened anxiety and prolonged and severe hypoactivity (amygdala), HPA-axis activation (paraventricular nucleus) and afebrile infections and exaggerated hypothermia (medial preoptic nucleus) (162–164).

### Conclusions

In this section, we have outlined the role of dysregulated inflammation as the driver or an influential factor for the most common chronic conditions affecting our communities. Inflammation has a systemic effect, as a universal response mechanism to protect the *person as a whole*. It is undiscerning of its origin or target cells/organs. While we readily recognize the effect of inflammation in acute disease processes, the uncontrolled or over production of inflammation leading to chronic low-grade effects are clearly under-recognized in clinical practice. Inflammation as the driver of most “phenotypically defined” disease also clearly challenges the prevailing notion of multimorbidity as a construct of “multiple individual diseases” in the one person (59, 165–173). Using the complex system's lens, inflammation is an inter-system communicator of distress as well as an indicator or target for addressing multiple chronic diseases.

## Implications for clinical practice

In the first part of this paper we reviewed the basic physiological mechanisms of inflammation and discussed to the pivotal role of the HPA-axis and the autonomic nervous system in modulating inflammation as a *whole body* response. The second part of the paper detailed the inflammatory mechanisms underlying some of the common diseases in the community, an aspect so far largely neglected in clinical practice. In this final part, we consider the implications of these detailed understandings on the future of clinical medicine, prevention and health system redesign, including community and public policy.

### Inflammation and the future of clinical medicine

The biomedical framework of the nineteenth century worked for the most common causes of disease and mortality in the 1800's and early 1900's. Historically, von Rokitansky's^3^ correlation of clinical symptoms of disease with their post-mortem anatomical changes (174) and Koch's^4^ discovery of bacteria as the causative agents of the major morbidities formed the basis for the development of the still prevailing biomedical framework of medicine. This framework states that *a specific cause results in a specific disease*. Unfortunately, in the twenty-first century, the biomedical approach to disease management is clearly no longer effective for the vast majority of chronic health conditions (175, 176).

Twentieth century research efforts have revealed that many common diseases share interrelated genomic and proteomic characteristics that define the *diseasome* (57). A diseasome represents varied genotypical and phenotypical characteristics that result in overlapping disease patterns (57, 177). At the molecular level, diseasomes are driven by interactions that themselves form highly complex dynamic intracellular and intercellular networks that in turn link all tissues and organ systems and their connections to the external environment. Hence, “so-called” specific disease can *no longer* be regarded as *discrete and independent from others* and rather need to be understood in terms of their underlying *complex adaptive system dynamics*.

As shown above the inflammatory cascades facilitate a person's systemic responses of balancing internal and external perturbations within their living environments. The *objective*, i.e., biomedically defined diseases, and the *subjective* of any illness, i.e., the experience of “dis-ease” (178, 179), arise from the *same* interdependent processes (1, 55, 59), thus any disease/illness *emerges simultaneously* from environmental, emotional, social and cognitive perturbations.

Given that community epidemiology consistently shows that only 4% experience conditions that require biomedical interventions (180–182) health professionals need to pay much greater attention to the person's external perturbing issues—housing, family dynamics, education, physical, and social environments etc.—i.e., apply the biopsychosocial/somato-psycho-socio-semiotic model to addressing health issues (178, 179, 183).

#### Determining overall inflammatory load

For clinicians and patients alike being able to monitor inflammatory load is helpful in managing disease and illness. Clinical markers like CRP are part of the acute-phase response to acute and chronic inflammation (184). CRP out-performs erythrocyte sedimentation rate in terms of responsiveness and specificity for inflammation. While elevated CRP is suggestive of inflammation or infection in the appropriate clinical context, it can also occur with obesity, renal dysfunction, cardiovascular disease, and many other chronic diseases. Conversely, a lack of C-reactive protein elevation in inflammation may be seen with hepatic failure, as well as during flares of conditions such as SLE. ESR measurements remain helpful in these and certain other clinical situations, such as the assessment of paraproteinemias (185), which often do not elicit an acute phase response. Table 2 outlines the clinically relevant inflammatory biomarkers and their interpretation. In addition, lymphocyte subset analysis can provide insight into the immune cell distribution and current state of the immune system (186).

**Table 2:** Markers of inflammatory activity.

**Marker**	**Normal range**	**Interpretation**
High sensitivity C-Reactive Protein (CRP) (median CRP of 0.8 mg/L, 90% of healthy people have CRP < 3.0 mg/L)	< 1 mg/L < 3 mg/L < 10 mg/L >10 mg/L	Low risk for CVD Moderate risk for CVD High risk for CVD very high risk for CVD or acute illness
Erythrocyte sedimentation rate (ESR)(mm/h)	Males: < 15 (< 50 y.o.) < 20 (≥50 y.o.) Females: < 20 (< 50 y.o.) < 30 (≥50 y.o.)	Faster sedimentation rate is associated with inflammatory conditions like infections, rheumatoid arthritis or cancer

Potential additional markers may include cytokines such as IL-6, TNF-α, and IL-1 as well as herpesvirus reactivation as indirect proxy of elevated inflammatory load. For example, individuals with IL-6 levels at or about 3.19 pg/mL were 2 times more likely to die than those with levels < 1.46 pg/mL (187). In general, cytokines like IL-6, TNF-α, and IL-1 levels follow a diurnal rhythm such that peak levels occur during the early night and reach a nadir in the morning (188, 189). This strong diurnal rhythm may explain why a clinical cutoff has not been identified. In an elderly population, herpesvirus reactivation was associated with elevated CRP and IL-6 (190), suggesting that an increase in antibodies to herpesviruses like EBV and CMV could be a useful marker of inflammatory load or a compromised immune system. Without a clinical cutoff, these markers would need to be taken multiple times to understand the natural variation within a person prior to utilizing it as a diagnostic marker of sustained inflammatory load.

#### Managing disease

Understanding the molecular/cellular level interactions of disease as fundamentally regulated by the cascades of inflammation broadens the understanding of disease causing mechanisms and thus widens the scope of managing disease in general terms. Whilst undoubtedly there is a need to deal with the “macroscopic” aspects of disease, be it infection, organ malfunction/failure or malignant disease, this alone is no longer sufficient. Our understandings now clearly indicate that “diagnosable disease” is the end-product of inflammatory processes; thus, our therapies primarily need to modulate all three components of immune function—the cognitive/experiential, neurological, and immunological, complemented by “disease-specific” interventions.

#### Managing illness

Recognized collections of symptoms can be grouped into syndromes, which represent examples of illnesses rather than pathophysiologically-defined diseases. Whilst such conditions are not amenable to simple *one cause-one disease* reduction according to the model provided by Koch's postulates, the utility of syndromes in clinical practice are well established. Take, for example, the illness of “fibromyalgia.” Suggesting to a patient with fibromyalgia “there is nothing wrong with you,” or worse “it's all in your head,” when unable to identify a “known disease” has always been poor clinical practice and clearly is no longer tenable in scientific terms. Psychoneuroimmunological-pathways have elucidated the interdependence between somatic and cognitive experiences—at a physiological level their interdependent pathways are one and the same (1, 23, 55, 191). Current fibromyalgia models suggest the presence of disordered pain transmission resulting in a state of “central pain sensitization,” although a range of parameters (e.g., sleep cycle disorders, autonomic dysfunction, HPA-axis dysfunction) are demonstrably disturbed, either as a primary or a secondary phenomenon. As up to 50% of fibromyalgia-sufferers have a life history of depression, one might expect an underlying inflammatory substrate like that described in the section above (depression).

Successfully managing the inflammatory state of illness requires three simple steps:

- First, help the patient to understand their underlying distress as the source of their illness experience;
- Second, engage the patient in reducing their systemic pro-inflammatory state through modification in diet, increased physical activity, and regular relaxation and meditative exercises; and
- Finally, symptom relief may at times require the short-term use of allopathic interventions.

### Prevention equals the reduction of pro-inflammatory activity

Given the role of inflammation in the pathogenesis and/or prognosis of illness, we require a change in our approaches to illness and disease prevention. Since inflammatory activity results in illness experience as well as producing definable diseases, successful prevention programs need to target those areas of the individual and his/her societal environment that promote pro-inflammatory stimulation.

#### Individual level anti-inflammatory prevention

Individual level prevention should start prenatally—enhancing and maintaining a mother's resilience—and span the length of an individual's life. Building physical resilience requires food intake free/low in inflammation provoking gut responses, plenty of physical activity to use up the energy provided by the physiological stress response (catecholamines and cortisol) relating to our daily interactions at home and work, and meditative activities to reduce brain and peripheral pro-inflammatory responses.

The old adage—*you are what you eat*—has deepened in meaning, given recent theoretical and technological advancements. A diet low in saturated fat and refined sugar and high in complex carbohydrates, fiber, protein from fish, healthy polyunsaturated fatty acids (PUFAs) from nuts, and fresh fruits and vegetables has long been touted as the gold standard, but evidence as to why was limited. Advancements in the gut microbiome have brought clarity (192–194). For example, the human digestive tract contains over 100 trillion symbiotic microbes that assist in digestion, affects the immune system and integrity of the intestinal wall, and influences brain function and behavior (195). The brain-gut axis hypothesizes that bacteria and their by-products from digestion regulate production of inflammation, neurotransmitters, neurotransmitter pre-cursors, and function of the enteric nervous system (195, 196).

The composition of the gut microbiome is now measurable (197), allowing the ready demonstration of food consumption changes on the composition of the gut microbiome (198–200). Complex carbohydrates and unsaturated fats support anti-inflammatory bacteria, while simple carbohydrates like refined sugar and less healthy fats such as omega-6 fatty acids bolster pro-inflammatory bacteria (201). Elevated inflammation in the gut has been linked to detriments in both physical and mental health (202, 203). Thus, discussion of a gut health and food selection of less processed or slow food is a critical step to intervention and prevention.

Physical activity, not vigorous exercise, is necessary and natural. Typically, toddlers and pre-school children have access to play time and being physically active. Once in primary school, recess or free time as well as physical education classes are often limited and leads to little to no physical activity in secondary education (204, 205). Leisure physical activity or participation in sports seem to be the extent of adolescents' involvement in physical activity and this time point is critical in establishing young adulthood activity levels (206). Unfortunately, this sedentary lifestyle is pervasive, as technological advancements have greatly reduced the need to be physically active in our everyday lives. Yet we have evolved from active ancestors including just a few generations ago (205, 207). The disconnect between how active we once were to today is astounding and contributing to major health issues related to “near unavoidable” excessive adipose tissue deposition (208). In addition to the lack of physical activity, the readily available, addicting, high-calorie food options offer a double whammy for weight management and systemic inflammation (209).

Leisure exercise or physical activity play a pivotal role in maintaining an individual's global health (210). Physiologically, the stress systems are acutely activated and support the physical exertion demanded to move the body through the physical activity, especially concerted exercise. This physical challenge allows the *SNS* and *HPA-axis* to activate and then stop once the exercise session is over (211). Following exercise, muscles release cytokines, known as myokines, to attract immune cells to repair the tissue damage caused by the exercise session (212, 213). Although there is an acute rise in inflammation, physically active individuals have lower systemic inflammation at rest compared to their sedentary counterparts (214, 215). Furthermore, exercise promotes microbiota diversity and overall gut health (216, 217). Bringing awareness to unfavorable outcomes associated with a sedentary lifestyle could encourage more physical activity—not just exercise—and prove critical to the prevention and intervention of physical and mental chronic health conditions.

Given technological advances such as smart phones, humans are often overstimulated and choose to reduce sleep to manage work and personal demands. This negative combination leads toward a shift in autonomic nervous system balance, favoring sympathetic activity and driving the body into a fight-or-flight state. To correct this problem, participating in self-care behaviors such as meditation, prayer, being outside, deep breathing, and focusing on the present moment or being mindful appear to stimulate the parasympathetic nervous system and restore autonomic balance (218, 219). In addition, physical activity or exercise can be thought of as a eustress; it activates the stress response systems, shuts them off following conclusion of the activity and enhances psychological well-being (210, 215, 220). Hence, physical activity and exercise may improve health because it trains the physiological systems to turn on and switch off, enhancing parasympathetic activity; resulting in the opposite of chronic stress where there is overactivation of the stress systems. Taken together, an individual can lead an anti-inflammatory lifestyle by disconnecting from electronics and living in the present while preparing meals with less processed foods and increasing their physical activity. While this conclusion is not groundbreaking, the struggle to get individuals to make these positive health choices is real and often not 100% within the person's control.

#### Environmental level anti-inflammatory prevention

When health care providers intervene, the patient is the focus, as health regularly is perceived to be under the individual's sole control. As outlined above health behaviors like food choice and physical activity engagement can directly influence the internal milieu, however, health is complex and influenced by many environmental factors outside the control of the individual (221).

We constantly interact with our physical and social environment that if experienced as constantly challenging, results in increased pro-inflammatory activity explaining the differences in morbidity and mortality patterns between socioeconomically privileged and disadvantaged communities. Overwhelming evidence supports the role of social determinants in an individual's overall health. For example, lower socioeconomic status (SES) has been associated with greater prevalence of cardiovascular disease, hypertension, obesity, type 2 diabetes, herpesvirus reactivation, and sero-positivity for multiple herpesviruses (i.e., greater viral load), shorter life expectancy, and mental health issues compared to their more financially well-off counterparts (222–227). Unfortunately, SES has many more factors than less financial security that can drive the health disparities that are observed. In sociodemographic terms, SES is also linked to less and reduced quality education, poorer and unstable living conditions, reduced neighborhood safety, exposure to greater violence, no or limited access to health care, decreased and/or insecure employment, and being of minority race or ethnic descent (228–236).

Health promotion under this perspective equates to community development and public policy as the greatest tools to reduce socioeconomic disparities and improve health. It was thought that SES may affect health via health care utilization due to many lower SES individuals not having any or enough healthcare insurance; however, even in countries with universal healthcare, the relationship between SES and health disparities can still be observed (237, 238). Thus, addressing health disparities requires this expansive, omniscient lens. There is a broad-based literature that has examined the mechanisms and impacts of these aspects of healthcare (228–236). Physicians and health care workers must be aware of the additional factors that influence their patient's health; the person sitting across of them is an agglomeration of factors: individual choice, work and living conditions, interpersonal relationships, family/cultural history, public policy, and socioeconomic environment.

#### Gene-environment interactions

Elucidation of the epigenome has reinforced the potent effect of our environment on bodily function—even gene expression. The best-defined gene-environment interaction in autoimmunity is the gluten (gliadin)-triggered inflammation seen in coeliac disease (239). In this condition of T-cell-mediated gut mucosal damage and secondary malabsorption, gluten-derived cleavage products produced by the action of the intestinal enzyme tissue transglutaminase 2 (tTG2) bind tightly to the pocket of the MHC molecule HLA-DQA1^*^0501:DQB1^*^0201 (corresponding to serotype HLA-DQ2), facilitating presentation of these peptides to gluten-specific T-cells, which then cause direct damage to the gut absorptive epithelium. This HLA haplotype is necessary but not sufficient for coeliac disease development, with its absence virtually excluding coeliac as a diagnosis, but with the allele (which confers a relative risk for coeliac disease of 7) also being seen in 20–30% of the healthy population (240). Another pertinent example of gene-environment interaction is the greatly increased risk of rheumatoid arthritis (RA) development in people with the HLA “shared epitope” DB1 in smokers vs. non-smokers (241). Additional important environmental modulators of autoimmune risk include local tissue damage [e.g., ischemia or trauma exposed previously hidden (“occult”) antigens], hormones, vitamin D sufficiency, UV light exposure, and various chemicals and drugs.

The association between autoimmunity and infective triggers is complex, with both protective and detrimental effects being described. On one hand, infection may trigger autoimmunity through either APC activation (expression of co-stimulation) or molecular mimicry (where part of the microbe resembles a self-structure, leading to autoimmunity). However, in contrast, infection may be protective, much like the protective effect seen against allergy: for example, the risk of diabetes in an at-risk mouse model (NOD mouse) rises substantially when the mouse is raised in a germ-free environment without the acquisition of a normal “microbiome” upon which the developing immune system can exercise itself (242). As discussed above with reference to this “Hygiene hypothesis,” evidence exists that changes in microbe-derived stimuli over the last three decades may have been partly involved in the epidemic of allergic and autoimmune conditions seen over this time—for example, the prevalence of inflammatory bowel diseases [IBD, (243)], diabetes (244) and multiple sclerosis (MS) have risen significantly (245).

Furthermore, in line with the stress, chronic disease, and inflammation lens, social factors such as loneliness and social support have an incredible impact on one's health. Pioneering work by Steven Cole has identified gene expression changes that occur in response to social stress (55). Social stress has been defined in a variety of ways including low SES, childhood adversity, social isolation, and social threat; these adverse events induce the conserved transcriptional response to adversity (CTRA) that elicits a pro-inflammatory environment and suppresses the immune system's antiviral activity and antibody production (246). Paucity of research links CTRA to chronic disease, however, some initial evidence suggests that mind-body interventions such as mindfulness and yoga can minimize the CTRA profile expression and may reduce systemic inflammation (247).

#### Pharmacological immune-directed therapy

Beyond health behaviors and socio-environmental context, immune function can be modified via a multiple of pharmacotherapies. Currently, in clinical practice, immune-directed therapies can be divided into three major classes, as shown in Table 3 and discussed below in more detail. However, accumulating evidence suggests that bioactive lipids including specialized pro-resolving mediators (SPMs) may offer new targets to manage chronic inflammation (49, 50, 54). SPMs are derived from omega-3 PUFAs (53), providing a mechanism underlying the relationship between lower systemic inflammation and a diet high in omega-3 PUFAs from fish and nuts. Furthermore, the endocannabinoid system's role in reducing inflammation especially neuroinflammation is becoming clearer (51, 54), but data are still limited and widespread clinical use has yet to occur.

**Table 3:** Immune modulating interventions.

	**Antigen-specific immunotherapy (“Desensitization”)**	**Immune modulation**	**Immunosuppression**
Characteristics	Increasing dose regular administration of pathogenic antigen, either subcutaneously or sublingually	Often derived from antimicrobial drug families	Often derived from chemotherapy and anti-rejection agents. Biologicals target specific cytokines (e.g., TNF, IL1)
Examples	Desensitization to dust mite, grass, animals, and molds	Hydroxychloroquine (Plaquenil), salazopyrine	Prednisolone, azathioprine, methotrexate, Infliximab (anti-TNF), Rituximab (Anti-CD20 [B cell])
Uses	Control of allergic symptoms in rhinitis and asthma	Amelioration of symptoms of autoimmunity (e.g., fatigue, rash, arthritis)	Control of severe systemic inflammation as well as steroid-sparing activity
Toxicities	Severe allergy uncommon with gradual schedules	Generally well-tolerated	Leucopenia, hepatotoxicity, secondary malignancies, infections
Unique Aspects	Potential for permanent deviation of immune response. Possible role in secondary prevention	No suppression of normal immune responses to organisms	Newer targeted biological therapies offered more focused immune suppression

##### Antigen-specific immunotherapy

In allergic disease where well-defined, relevant antigens are identified as causing ongoing inflammation, specific immunotherapy, often referred to as “desensitization,” can play a valuable role (248). Where inhalant allergy (e.g., house dust mite, grass, animal, mold) contributes to allergic rhinoconjuctivitis (colloquially referred to as “hay fever”) or asthma, a course of allergen delivery through non-respiratory routes for prolonged time-periods can induce a “switch” from T_H_2 to T_H_1 deviation (and upregulation of T_REG_ cell activity). Following adequate treatment duration (often 2–3 years), the immunological change and inflammatory abatement can be permanent.

##### Immunomodulation

A unique member of this class of therapy, which reduces autoimmune tendencies without compromising host antimicrobial activity, is hydroxychloroquine (“plaquenil”). This antimalarial drug is believed to act upon antigen-presenting cells to reduce their propensity to present and/or be activated by self-antigens, hence reducing inflammatory symptoms (e.g., rash, arthritis, fatigue) associated with systemic autoimmune conditions such as lupus and Sjogren's (249). Other agents traditionally employed as antimicrobials also offer valuable immune-modulating effects, such as salazopyrine (especially for rheumatoid arthritis), dapsone (for cutaneous lupus), and macrolides (for allergic and other forms of chronic bronchitis).

##### Immunosuppression

Whilst being the “bluntest” and most toxicity-prone, immune-suppressive agents are called upon when organ function is threatened by systemic inflammation. Corticosteroids (e.g., prednisolone, hydrocortisone) offer rapid anti-inflammatory activity and, at high doses, can also induce apoptotic death of autoimmune pathogenic lymphocytes. Unfortunately, these potentially life-saving benefits cannot be disentangled from the multiple toxicities associated with long-term or high-dose steroid treatment, including hypertension, osteoporosis, dyslipidemia, and diabetes. In order to reduce the required steroid dose whilst still maintaining inflammatory control, the so-called “second-line,” “disease-modifying” or disease-modifying antirheumatic drug (DMARD) agents are employed. Many of these drugs were developed in a cancer or transplant rejection setting, but have displayed useful activity in a range of systemic autoimmune conditions, such as SLE and RA (250). More recently, anticytokine therapies (the so-called “biologicals”) have provided a degree of greater selectivity in terms of focused immune suppression, although infections are still a recognized and common problem with these agents (251).

### Health system design challenges—creating anti-inflammatory living conditions

A health focused health system is one that purposefully addresses factors at all levels affecting the health and well-being of all individuals in the entire community (252). In policy terms this requires the understanding that all agents interact with each other and that overlooking these interactions can easily result in undesirable and unforeseen outcomes (253). Hence, it is important to consider that besides of contemplating the health service delivery environment, one also needs to focus on all of the external environmental factors affecting a person's life, education, housing, work, social, and public infrastructure.

#### Health service delivery

Health service delivery urgently needs to adopt a truly “patient needs” focus (252). Such needs are not limited to the biomedical components of the patient's illness but include aspects that contribute to his illness experience ranging from personal to home and community constraints. *Health-focused* service delivery is a team effort that involves as much health professionals as social and community workers all of whom share the elementary responsibilities of alluding to illness-promoting factors (pro-inflammatory) and engaging in their remediation (anti-inflammatory) (59, 254).

#### External environmental factors

During our evolution, the external environment demanded constant vigilance for survival; any potential threat resulted in the physiological stress response—a rise in epinephrine, norepinephrine, and cortisol with the associated rise in blood pressure, heart rate and mobilization of glucose—to allow us to flee from danger and ensure or ongoing survival. These “hard wired survival responses” remain active even though our environments have little in common with that of our early forbearers. Today's threats arise as much or more from our built environment, including housing, neighborhoods, artificial light, and public infrastructure that minimizes physical activity than interpersonal threats to our survival, but these physical environmental stressors receive little attention. Furthermore, our social world and its growing diversity can introduce demands that our brains have limited experience with such as overcrowding, school and work environments, electronic communications/interactions, and insufficient social infrastructure (59, 231, 254, 255). Healthcare professionals have limited control over the sociocultural and environmental decisions; however, it is critical that they embrace their social responsibility, as Virchow exemplified, to educate and to bring awareness about these aspects for maintaining and regaining health to patients and elected politicians alike (256).

## Conclusions

Chronic, non-communicable diseases plague individuals' lives and the healthcare system, financially burdening society (252). The chronic diseases covered above involve a variety of organ systems, yet excessive inflammation is a common thread in all forms of disease development and progression. Chronic stress dysregulates immune function via the hyperactivation of the SNS and HPA axis and withdrawal of the PNS shifting lymphocytes into a pro-inflammatory state with reduced anti-inflammatory influence (55, 257). Thus, prioritizing *anti-inflammatory lifestyle* should be a primary approach for both prevention and intervention.

An individual's health evolves out of complex interactions among sociocultural, public policy, physical environment, interpersonal, and intra-individual physiological levels (59). The physiological stress of one system failing plus psychological stress from the environment proves a potent combination that forces the dysregulation of additional systems, leading to multimorbidity, a dynamic interplay largely ignored in the current medical teaching and practice. There is an urgent need for healthcare professionals to migrate from the single organ system perspective to recognizing that the development of a disease is a signal that the patient's body is in a state of multiple system dysregulation. This shift requires a multi-pronged response to promote anti-inflammatory interventions at the lifestyle, coping and physiological levels.

A transdisciplinary team approach to patient care would aid a patient to develop and sustain an anti-inflammatory lifestyle. For example, a patient with their first persistently mild to moderately elevated blood pressure readings or just out of range blood sugar readings should alert health professionals to the potential of chronic *SNS* and *HPA*-axis activation and *PNS* withdrawal, prompting a discussion about psychological distress, nutrition and physical activity. If the screening indicates high distress, the patient should—in an ideal world—be connected with an in-house health psychologist or counselor. If poor diet or physical activity behaviors are noted, then the patient should be directed to an integrated nutritionist or health coach. This integrative team needs a lead physician, one with a diverse background like primary care or general internist, to merge and synthesize the patient's distress as a *whole of person* response.

Although the health care field can directly intervene with the individual, an individual's health is influenced by many factors outside of personal choice such as public policy, physical structure of their environment, crime in their neighborhood, sociocultural norms, and socioeconomic status. Hence, healthcare professionals must become advocates for social change and improving their patients' environment in a more societally impactful way by engaging in community wellness events, leading seminars for laypeople, and educating politicians and policy makers (252).

Given the unprecedented role that chronic inflammation plays in the development of and the recovery from physical and mental disease, healthcare professionals, and researchers must educate patients and laypeople to seek anti-inflammatory preventive and therapeutic interventions. At an individual level, we must provide guidance on better food choices, engagement in less sedentary behaviors, and practice stress management skills to activate the parasympathetic nervous system and prioritize sleep. At a societal level, we need to be advocates for infrastructure changes and break down socioeconomic disparities (252).

## Author contributions

All authors reviewed and approved the final version of the manuscript. JPS conceived the paper, pulled together the team, created the images and was the primary writer of the coronary artery disease, osteoarthritis and aging sections and drafted the initial version of the implications for clinical practice section. JMB assisted in the theory development, drafted first versions of part 1, was the primary writer of the obesity, insulin resistance and type-2 diabetes, and depression sections as well as socioecological perspectives in clinical care sections, and the overall conclusions. GEB was primary writer of cardiac arrhythmia sections. GR was primary writer of atopy, asthma, and allergic rhinitis and autoimmunity sections and drafted the immunology treatment section. JMB was responsible for readying the manuscript for journal submission and publication as corresponding author.

### Conflict of interest statement

The authors declare that the research was conducted in the absence of any commercial or financial relationships that could be construed as a potential conflict of interest.

## Footnotes

^1^Diseasome describes the combined set of all known disorder/disease-gene associations.

^2^Often using the “CAP” technology that has superseded previous radioactive “RAST” tests.

^3^Carl Freiherr von Rokitansky (1804-1878), a Vienna pathologist, father of the clinico-pathological correlation.

^4^Robert Koch (1843-1910), German microbiologist, father of the germ theory of disease.

## Abbreviations

α: alpha
AAD: allergic airway diseases
ACTH: adrenocorticotropic hormone
AF: atrial fibrillation
AhR: aryl hydrocarbon receptor
AIRE: autoimmune regulator gene
ANA: anti-nuclear antibodies
Anti-CCP: anti-cyclic citrullinated peptide
AP-1: activator protein-1
APC(s): antigen presenting cell(s)
β: beta
BMI: body mass index
CCL: C-C chemokine ligand
CD: cluster of differentiation
CTL: cytotoxic T lymphocytes
CMV: Cytomegalovirus
CNS: central nervous system
COX-2: cyclooxygenase-2
CRH: corticotropin releasing hormone
CRP: C-reactive protein
CTRA: conserved transcriptional response to adversity
CXCL: C-X-C chemokine ligand
DAMPs: damage-associated molecular patterns
DC(s): dendritic cell(s)
DMARD(s): disease-modifying antirheumatic drug(s)
EBV: Epstein-Barr virus
γ: gamma
HLA: human leukocyte antigen
HLA-DR: human leukocyte antigen class II
HPA: hypothalamic-pituitary-adrenal
HRV: heart rate variability
IBD: inflammatory bowel diseases
IFN: interferon
Ig: Immunoglobulin
IL: interleukin
IPEX: a Immune dysregulation polyendocrinopathy enteropathy X-linked syndrome
IRF: interferon-response factors
LDL: low-density lipoprotein
LPS: lipopolysaccharides
LT: leucotrienes
MHC: major histocompatibility complex
MMP(s): matrix metalloproteinase(s)
MS: multiple sclerosis
nAChR: nicotinic acetylcholinergic receptor
NF-κB: nuclear factor – kappa B
NK: natural killer
NSAID(s): non-steroidal anti-inflammatory drug(s)
PAMPs: pathogen-associated molecular patterns
PGE2: prostaglandin E2
PNS: parasympathetic nervous system
PRR(s): pattern-recognition receptors
PTH: parathyroid hormone
PUFA(s): polyunsaturated fatty acid(s)
RA: rheumatoid arthritis
ROS: radical oxygen species
SES: socioeconomic status
SLE: systemic lupus erythematosus
SNS: sympathetic nervous system
SPM(s): specialized pro-resolving mediator(s)
T_C_: T cytotoxic cells
TCR: T cell receptor
TGF: transforming growth factor
T_H_: T helper cells
TIMP: tissue inhibitor of metalloproteinases
TLR(s): toll-like receptor(s)
TNF-α: tumor necrosis factor-alpha
T_REG_: T regulators cells
TSH: thyroid stimulating hormone
tTG: tissue transglutaminase
VZV: Varicella Zoster virus.
